# *SgPAL1/2* confers anthracnose resistance in *Stylosanthes guianensis* by modulating lignin content and monomer ratios

**DOI:** 10.1186/s12870-025-07720-2

**Published:** 2025-12-24

**Authors:** Mengze Gao, Xipeng Ding, Yajun Jiang, Ronghui Dai, Jianyu Zhang, Pandao Liu, Guodao Liu, Lingyan Jiang, Lijuan Luo

**Affiliations:** 1 School of Breeding and Multiplication (Sanya Institute of Breeding and Multiplication), School of Tropical Agriculture and Forestry, Hainan University, Sanya, Hainan province China; 2 Tropical Crops Genetic Resources Institute, Chinese Academy of Tropical Agricultural Sciences, Haikou, Hainan province China

**Keywords:** Legume forage; *Stylosanthes Guianensis*, Anthracnose, Lignin content, G/S ratio of lignin monomers

## Abstract

**Background:**

Anthracnose, caused by the hemibiotrophic fungus *Colletotrichum gloeosporioides*, is one of the most destructive diseases affecting leguminous forage crops worldwide. Due to its broad host range and ability to cause severe yield losses, identifying genetic resistance is crucial for sustainable disease management. *Stylosanthes guianensis*, a diploid, self-pollinating tropical legume widely cultivated as forage, cover crop, and green manure, exhibits natural variation in anthracnose resistance, making it an ideal model for investigating host resistance genes.

**Results:**

In this study, we evaluated the resistance of 28 *S. guianensis* accessions to *C. gloeosporioides*, revealing a significant positive correlation between phenylalanine ammonia-lyase (PAL) activity and anthracnose resistance. Transcriptome profiling identified five *SgPAL* genes, with *SgPAL1* and *SgPAL2* showing particularly strong induction upon pathogen infection and correlation with PAL activity. Functional characterization confirmed these genes encode cytoplasmically localized PAL enzymes. Overexpression of *SgPAL1/2* in *Arabidopsis thaliana* enhanced resistance to *C. gloeosporioides*, while integrated transcriptomic, metabolomic, and biochemical analyses demonstrated that *SgPAL1/2*-mediated resistance operates through upregulation of lignin biosynthesis pathways, resulting in increased total lignin content and altered guaiacyl (G)/syringyl (S) monomer ratios.

**Conclusion:**

This study identified *SgPAL1/2* as crucial genetic regulators of lignin-mediated anthracnose resistance. Our findings further revealed that the increase in both lignin content and the G/S ratio are significant factors enhancing resistance, offering promising targets for the genetic improvement of forage legume crops.

**Supplementary Information:**

The online version contains supplementary material available at 10.1186/s12870-025-07720-2.

## Background

Anthracnose, caused by hemibiotrophic fungal pathogens of the genus *Colletotrichum*, represents one of the most destructive diseases affecting leguminous forage crops worldwide [1, 2]. This devastating pathogen not only causes substantial yield reductions and forage quality deterioration but also contributes to progressive degradation of grassland ecosystems, posing significant challenges to sustainable pasture management [2]. The pathogen’s sophisticated infection strategy involves an initial biotrophic phase characterized by stealthy colonization of host tissues, followed by a destructive necrotrophic phase leading to tissue maceration [3, 4]. Further complicating disease management, *Colletotrichum* species exhibit an exceptionally broad host range (>760 plant species) and rapidly develop fungicide resistance, rendering conventional chemical control strategies increasingly ineffective [5, 6].

The development of host plant resistance remains the most promising and sustainable approach for anthracnose management. However, genetic resources for resistance breeding remain severely limited. To date, only two anthracnose resistance genes have been functionally characterized in leguminous forages: *RCT1*, a canonical TIR-NBS-LRR resistance gene from *Medicago truncatula*, and *ATL31*, a plasma membrane-localized E3 ubiquitin ligase from *Stylosanthes guianensis* [7, 8]. Although both genes confer broad-spectrum resistance against *Colletotrichum* when transferred into susceptible alfalfa (*Medicago sativa*) or *Arabidopsis thaliana*, their long-term effectiveness is compromised by the ongoing co-evolutionary arms race between pathogens and host defense systems, an evolutionary dynamic that frequently results in resistance breakdown [6, 9]. These limitations highlight the critical need for continuous identification and characterization of novel resistance genes to ensure durable protection of leguminous forage crops against this rapidly adapting pathogen. The discovery of additional resistance mechanisms will not only expand our fundamental understanding of plant-pathogen interactions but also provide essential genetic resources for developing anthracnose-resistant forage varieties through modern breeding approaches.

Central to many plants’ defense arsenals is the phenylpropanoid pathway, which generates diverse protective compounds including structural polymers like lignin and antimicrobial phenolic metabolites [10]. Phenylalanine ammonia-lyase (PAL) serves as the gateway enzyme to this crucial pathway, converting phenylalanine to cinnamic acid in the rate-limiting first step [11]. Research across multiple crop systems has consistently demonstrated PAL’s pivotal role in plant immunity. In rice, specific PAL isoforms enhance resistance to the brown planthopper through coordinated activation of both lignin biosynthesis and salicylic acid signaling pathways [12]. Similarly, soybean PAL genes have been shown to confer resistance against *Phytophthora* pathogens by upregulating key enzymes in the lignin synthesis cascade [13]. The conservation of PAL-mediated defense mechanisms across diverse plant species, from cereals to legumes, highlights its fundamental importance in plant-pathogen interactions while suggesting opportunities for translating knowledge between systems. However, despite its established role in model crops, PAL-mediated resistance in tropical legumes remains underexplored, particularly in the context of anthracnose.

*S. guianensis* (stylo) is an important tropical forage legume valued for its remarkable adaptability to challenging environmental conditions, including drought, aluminum toxicity, and nutrient-poor soils [14–16]. As a diploid (2n = 20) self-pollinating species, stylo presents significant advantages for genetic studies compared to polyploid forage crops. Its simple genome architecture minimizes complications from genetic redundancy while facilitating the development of uniform genetic materials, features that make it particularly suitable for resistance gene identification and characterization [17, 18]. The molecular cloning of anthracnose resistance genes from stylo holds dual significance: it enables marker-assisted breeding for enhanced disease resistance within this species while simultaneously providing valuable genetic resources for improving related forage legumes with more complex genomes. Such discoveries would represent a major advancement in precision breeding approaches to address anthracnose, a particularly devastating disease in legume forage production systems.

Modern functional genomics tools and association genetics approaches have revolutionized our ability to analyze complex traits like disease resistance in non-model crops. Well-designed core collections that capture maximum genetic diversity have successfully identified resistance loci in various crops [19–21]. Our preliminary studies with *S. guianensis* cv. Reyan No. 2 demonstrated significant PAL enzyme activation following pathogen infection, suggesting its involvement in anthracnose resistance mechanisms [3]. However, key questions remain unanswered regarding which specific PAL gene family members mediate this defense response, their regulatory dynamics, and their effects on downstream defensive metabolites in the stylo system. Addressing these knowledge gaps is essential for developing effective molecular breeding strategies to achieve durable anthracnose resistance.

This study was designed to elucidate the molecular basis of PAL-mediated anthracnose resistance in *S. guianensis* through an integrated approach combining resistance phenotyping, functional genomics, and biochemical characterization. Specifically, it not only explored the function of *SgPALs* in disease resistance but further revealed the dynamic changes in lignin deposition during the defense response in *Stylosanthes*, underscoring the potential critical role of lignin monomeric reprogramming. Our findings demonstrate that increased lignin content and a raised G/S ratio are significant resistance mechanisms. These insights enhance the fundamental understanding of phenylpropanoid-mediated defense in tropical legumes and provide practical molecular tools for breeding programs.

## Materials and methods

### Plant material and growth conditions

The 28 accessions of stylo (*S. guianensis*) used in this study were derived from a core collection, representing the genetic diversity of 237 *S. guianensis* germplasms bank through integrated phenotypic and SSR genotyping analyses (The details are in Supplementary Material 3). All 237 accessions of *S. guianensis* germplasm used in this study were provided by the Tropical Crops Genetic Resources Institute & National Key Laboratory for Tropical Crop Breeding, Chinese Academy of Tropical Agricultural Sciences (CATAS). The repository information for these accessions is provided in Table S1. The seeds were soaked in hot water (80 °C) for 3 min, followed by a 3-day germination phase as previously described. Seedlings were then relocated to pots containing vermiculite and organic soil (2:1, v/v), watered by sufficient half-strength Hoagland’s nutrient solution, and grown under greenhouse condition (28 °C) for 30 days [22].

Ecotype Colombia (Col-0) and constructed transgenic *Arabidopsis* thaliana seeds were vernalized in sterile water at 4 °C for 2 days. Plants were then grown in a mixture of vermiculite and organic soil (2:1, v/v) under a 16-h light/8-h dark photoperiod at 22 °C.

### Fungal culture and preparation of spore suspensions

The *C. gloeosporioides* DZ-19, which co-infects *Arabidopsis* and stylo, was cultured on potato dextrose agar (PDA) for about 8 d at 28 °C. In 200 mL CM liquid medium, 4–5 pieces of round dics with diameter of 1 cm taken from the above dishes were added and incubated at 28 °C with 200 rpm shaking for 3 days. The spores were resuspended in sterile distilled water after double gauze filtration, and the spore concentration was adjusted with a hemacytometer.

### Evaluation of core collection for resistance to *C. gloeosporioides*

Twenty-eight accessions of *S. guianensis* were used in this study to evaluate their resistance to *C. gloeosporioides*. The method proposed by Wang was used to grow the plants [3]. Briefly, seeds were germinated for 3 days and transferred to soils to grow for 30 days before inoculation. One-month-old stylo plants were sprayed with spore suspensions of *C. gloeosporioides* DZ-19 containing 0.02% Tween 20 for evaluation of disease resistance. The spore suspension concentration was (1 to 2) × 10^7^ conidia/mL. Referring to Chakraborty’s study [23], five indicators of disease were assessed 4 or 7 days after inoculation: leaf disease severity (LRAT), stem disease severity (SRAT), defoliation (DEFL), Area of lesions/leaves (ALES), and dry weight index (DRWT). The degree of LRAT and SRAT was categorized into 6 levels: 0 = no visible disease, 1 = 0–1% tissue necrotic, 2 = 1–10% tissue necrotic, 3 = 10–25% tissue necrotic, 4 = 25–50% tissue necrotic, 5 = 50–100% tissue necrotic. ALES was measured with ImageJ (https://imagej.net/). DRWT indicates percent dry weight reduction. The disease index was calculated as follows:

$$\mathrm{disease}\;\mathrm{index}=\sum X_i\;Y_i/\left({\mathrm X}_{max}\cdot\sum Y_i\right)\times100\%$$

where *X*_*i*_ represents the disease grade value, and *Y*_*i*_ corresponds to the number of samples at grade *X*_*i*_.

LRAT, SRAT, DEFL, ALES were assessed from 15 plants, while DRWT were assessed from 5 plants from each accessionwith each measurement four times over two years. The 28 stylo were systematically clustered and analyzed by calculating the mean disease index of the five indicators pooled from the three replicates, using the Ward clustering method of IBM SPSS version 20 (Chicago, USA) and the heatmap cluster analysis of TBtools (Guangdong, China). The disease resistance index (LRAT’, SRAT’, DEFL’, ALES’, DWRT’) was calculated as follows:

$$\begin{aligned} \mathrm{disease}\;\mathrm{resistance}\;\mathrm{index}=&1-\sum{\mathrm X}_{\mathrm i}\;{\mathrm Y}_{\mathrm i}\\&/\left({\mathrm X}_{max}\cdot\sum{\mathrm Y}_{\mathrm i}\right)\\&\times100\% \end{aligned}$$

### Determination of PAL enzyme activity and correlation analysis with disease indexes

After inoculating the 28 accessions, leaves were collected at various time points (0 hpi, 24 hpi, 48 hpi, 60 hpi) in liquid nitrogen. The PAL activity in the plant leaves before and after inoculation was detected according to the instructions provided in Phenylalnine Ammonia-lyase Kit (A137-1, Nanjing Jiancheng Bioengineering Institute, Nanjing, China). The protein concentration was measured according to Bradford method [24]. Enzyme activity was given as unit (U) per milligram of protein. One unit was defined as a 0.1 change in absorbance at 290 nm per gram of leaf in 1 mL reaction system for 1 min. The formula for calculating PAL activity (U/mgprot) is given by:

$$\begin{aligned} \mathrm{PAL}\;\mathrm{activity}\;&\left(\mathrm U/\mathrm{mg}\;\mathrm{protien}\right)=\triangle\mathrm A290/0.1\\&/\left(Cpr\mathit\cdot\mathit\;Vs\right)\cdot\mathrm{Vt}/T \end{aligned}$$

where *Cpr* represents the protein concentration of the sample, *Vs*, *Vt* and *T* denote the volume of the sample used, the total volume of the reaction system, and reaction time respectively.

The linkET R package (version 0.0.3) was used to analyze the correlation between PALase activity and the disease resistance index in leaves [25].

### Identification of the *PAL* genes of stylo and analysis of gene structure, conserved motifs, and phylogenetic relationship

Information on PAL proteins in *Arabidopsis* was retrieved from The *Arabidopsis* Information Resource (www.Arabidopsis.org). The CDS information of PAL genes was obtained from the time-course transcriptomic data in *S. guianensis* after *C. gloeosporioides* infection (NCBI SRA accession number: SUB8868028). The protein sequences of stylo were compared to the seed sequences of *Arabidopsis* using BLASTP (version 2.9.0) with an e-value cutoff of 1e^− 10^ [25]. Candidate sequences were identified as those with at least 35% identity and at least 35% coverage. Lyase_aromatic structural domain information (PF00221) was downloaded from the Pfam database (http://pfam.xfam.org/). A total of five SgPALs were obtained after removing redundant sequences using the Reduction of redundancy tool (web.expasy.org/decrease redundancy). Sequence data of *SgPAL* genes from this study can be found in the database of NCBI under the following accession numbers: PQ788608 (*SgPAL1*), PQ788609 (*SgPAL2*), PQ788610 (*SgPAL3*), PQ788611 (*SgPAL4*), PQ788612 (*SgPAL5*).

CDS structures of the five *SgPAL* gene family members were analyzed using the online website GSDS (http://gsds.gao-lab.org/). Conserved motifs within the full-length PAL protein sequences were elucidated using the MEME online tools (http://meme-suite.org/). The PAL protein sequences from other species, downloaded from NCBI, were aligned with SgPAL protein sequences by Clustal W (Table S2). Phylogenetic analysis was conducted using the Neighbor-Joining method in MEGA 7 [26]. The bootstrap was set as 1000 replicates, and the tree was drawn to scale where the length of branch was proportional to the evolutionary distances. The tree file was visualized with iTOL (https://itol.embl.de/).

### qRT-PCR analysis

RNA from the sample was isolated using Trizol Reagent (Tiangen Biotech, Beijing, China), and reverse transcribed to cDNA using the HiScriptII 1 st Strand cDNA Synthesis Kit (Vazyme, Nanjing, China) following the product instructions. The qRT-PCR analysis was performed using SYBR Green Premix Pro Tag HS qPCR Tracking Kit (Rox Plus) (Accurate Biotechnology (Hunan) Co., Changsha, China) and a QuantStudio 6 Flex qRT-PCR system (Applied Biosystems, MA, USA). The qRT-PCR primers are listed in Table S3. Reaction mixtures of 10 µL contained 5 µL of 2X SYBR Green Pro Taq HS Premix, 0.25 µL of Forward primer (10 µM), 0.25 µL of Reverse primer (10 µM), 1 mL of cDNA template, and 3.6 mL of ddH_2_O. The amplification conditions were set at 95 °C for 10 min, followed by 40 cycles of 95 °C for 10 s and 60 °C for 30 s. For calculating gene expression in stylo and *Arabidopsis*, the reference genes *SgUBCE1* (Unigene24749_SG), *SgRPL19* (CL1636.Contig1_SG) and *AtACT2* (GeneBank accession name: AT3G18780) were used as internal controls, respectively [27]. Three independent experiments were performed. A series of two-fold dilutions of cDNA samples (1, 1/2, 1/4, 1/8, 1/16, 1/32, 1/64, 1/128) were used as templates to establish standard curves. Expression levels were calculated using the double standard curve method [28].

### Purification and biochemical characterization of SgPAL1 and SgPAL2

The ORFs of *SgPAL1* and *SgPAL2* genes were obtained by amplification using primers specific for pGEX-*SgPAL1/2* -F/R (Table S3). The resulting products and pGEX6P-3 vector were ligated with OK Clone DNA Ligation Kit (Accurate Biotechnology (Hunan) Co., Changsha, China). Subsequently, the recombinant plasmid with a GST tag at the N-terminus was transferred into *E. coli* BL21 Chemically Competent Cell (Weidibio, Shanghai, China). Expression of the fusion proteins was induced in *E. coli* with 150 µM IPTG. SgPAL1 and SgPAL2 proteins were purified using GST magnetic bead and L-Glutathione reduced elution (Solaibio, Beijing, China) with reference to BeaverBeads™ GSH instructions (Beaver, Suzhou, China). To test the protein purification, protein samples (20–30 mg) were separated by 10% SDS-PAGE and visualized after staining with Coomassie Brilliant Blue R-250 (0.1% w/v CBB R-250 in 10% v/v acetic acid and 40% v/v methanol). For immunoblot, the proteins were transferred to PVDP membrane for 2 h at 65 V on ice, and incubated with the primary antibody anti-GST (Cell Signaling and Technologiess, Danvers, USA). Chemiluminescent detection was performed using horseradish peroxidase-linked goat anti-rabbit antibody (Cell Signaling and Technologiess, Danvers, USA). Then, protein size on the PVDF membrane was observed using Coomassie Brilliant Blue R-250 staining (0.1% w/v CBB R-250 in 10% v/v acetic acid and 40% v/v methanol) with reference to the ColorMixed Protein Marker (11-180KD) (Solaibio, Beijing, China). Furthermore, the PALase activity of purified SgPAL1 and SgPAL2 proteins was detected using the Phenylalnine Ammonia-lyase Kit (A137-1, Nanjing Jiancheng Bioengineering Institute, Nanjing, China).

### Subcellular localization

The CDS regions of *SgPAL1* and *SgPAL2* genes were amplified from cDNA of Reyan No.5 stylo leaf using Phanta Max Master Mix (Vazyme, Nanjing, China). The amplified sequence was gel purified by FastPure Gel DNA Extraction Mini Kit (Vazyme, Nanjing, China) and cloned into the pA7-GFP plasmid. The correctly sequenced recombinant plasmid was transferred into *Agrobacterium tumefaciens* strain GV3101.

The protoplasts were isolated from cotyledons of 20-day-old stylo seedlings, which were treated in enzymolysis solution (1.5% cellelase R-10, 1.25% macerozyme R-10, 0.5 M mannitol, 4 mM MES) for 16 h. The protoplasts were then resuspended in MMG solution (0.4 M Mannitol, 15 mM MgCl_2_, 4 mM MES, pH = 5.7) until the concentration was 6 × 10^5^ cells/mL. The pA7::SgPAL1 plasmid were transferred into the 100 µL protoplasts under the facilitation of PEG (0.2 M Mannitol, 0.1 M CaCl_2_, 50% PEG-4000), and transformed for 5 min before being cultured in W5 (154 mM NaCl, 125 mM CaCl_2_, 5 mM KCl, 2 mM MES, pH = 5.7) for 16 h. Observation was made under a confocal microscope (A1RHD25 + N-SIM + N-STORM, Nikon) at 488 nm and 587 nm.

*Agrobacterium tumefaciens* containing pA7-GFP-SgPAL1/pA7-GFP-SgPAL2/pA7-GFP and pCXSN-mCherry plasmids were mixed in a 1:1 ratio and suspended in the buffer (10 mM MgCl_2_, 10 mM MES, pH = 5.7) with final OD_600_ value of 1.0. The suspension was infiltrated into the leaves of four-week-old *N. benthamiana* plants. After 2 days, leaf tissues were collected to detect GFP and mCherry fluorescence signals using a confocal microscope at 488 nm and 587 nm.

### Analysis of the functional properties of *SgPAL1* and *SgPAL2* in *Arabidopsis*

The CDS regions of *SgPAL1* and *SgPAL2* were amplified from the cDNA of Reyan No.5 stylo (Table S3). Recombinant plasmids Myc::SgPAL1 and Myc::SgPAL2 were constructed by linearizing the pCXSN-Myc vector with *XcmI* and then ligating the gene fragments with T4 DNA Ligase (Sangon Biotech, Shanghai, China). Overexpression plants were T3 generation stable plants obtained by transfection of the plasmid into Col-0 *Arabidopsis* by the flower dip method followed by generation-by-generation screening with MS solid medium (MS Salt, 1% sucrose, 0.75% phytogel) containing 30 µg/mL Hygromycin B (BioFroxx, German). The positive transgenic plants verified by both qRT-PCR and western blot were used for pathogen assays. The detached leaves of Col-0 and OE-*SgPAL1/2* lines were inoculated by dripping 10 µL conidial suspension (5 × 10^5^ conidia/mL) in the center. Photographs were taken after 6 days of incubation at 22 degrees Celsius, and the lesion area was measured using Image J (https://imagej.net/) (relative area of disease = lesion area/leaf area). The percentage of lesion area (lesion area/leaf area) was counted on 18 leaves per replication and pooled from three biological replicates. Meanwhile, the contents of fungal DNA in 100 ng of *Arabidopsis* leaf at 0 h and 60 h post-inoculation were detected by Q-PCR [29]. Data represent three independent biological experiments with similar results (*n* = 6).

### Microscopic observation of *C. gloeosporioides* infection in *Arabidopsis* leaves

*Arabidopsis* leaves were collected at different time points after inoculation and fixed in formalin acetic acid (FAA) solution containing endoethanol-formaldehyde-acetic acid (18:5:5, vol/vol). The samples were decolorized by immersion in saturated chloral hydrate solution (macalin) for 3 h, and then stained in 0.5% aniline blue solution for 15 min. The samples were rinsed with sterile distilled water and then observed under a light microscope (TL3201, Shanghai Thalon Optical Instruments Co., Ltd., China).

### Transcriptome analysis of *SgPAL1* and *SgPAL2* OE lines post inoculation of *C. gloeosporioides*

RNA sequencing was performed by Metware Biotechnology Co., Ltd. (Wuhan, China). The RNA was extracted by ethanol precipitation and CTAB-PBIOZOL. Total RNA was identified and quantified using a Qubit fluorescence quantifier and a Qsep400 high-throughput biofragment analyzer. Subsequently, mRNA was fragmented, and then used for synthesizing double stranded cDNA. End repair, sequencing adapter ligation, and PCR amplification enrichment were conducted to construct cDNA libraries. Finally, the qualified and quantified libraries were subjected to PE150 sequencing on the Illumina HiSeq platform. The raw Illumina reads generated from RNAseq experiments were deposited at NCBI SRA under the accession number: PRJNA1199688. The adaptor sequences and low-quality reads were removed from raw data to generate the clean data. HISAT2 (version 2.2.1) was used to build an index, and clean reads were aligned to the reference genome (Ensemble number: release-56). Genes were quantified using the FeatureCounts software (version 2.0.3).

The transcript abundance of each gene was calculated by the fragments per kilobase million mapped reads (FPKM) value using RNA-seq data. The changes in gene expression between 60 hpi and 0 hpi were computed using DESeq2. Genes with a minimal 2-fold difference in expression (|log_2_ fold change| ≥ 1) and FDR < 0.05 were considered as differential expressed genes (DEGs), and these DEGs were used for the following Kyoto Encyclopedia of Genes and Genomes (KEGG) and pathway enrichment analysis. Heatmaps on the expression patterns of the DEG were presented by TBtools.

### Measurement of lignin

Leaves of *Arabidopsis* and stylo before and after inoculation were collected, and the total lignin content was assayed by Lignin Content Assay Kit (BL893A, Biosharp, China). The absorbance of the solution at 280 nm was determined using Infinite 200 pro (TECAN, Swiss Confederation). According to the standard curve equation, the lignin content was calculated using the following formula:

$$\begin{aligned} \mathrm{Lignin}\;\mathrm{content}\;\left(\mathrm{mg}/\mathrm g\right)=& \left(\triangle\mathrm A280+0.003\right)/10.615 \\& \times\;\mathrm{Reaction}\;\mathrm{volume}\;\left(\mathrm{mL}\right)\\&/\mathrm{dry}\;\mathrm{weight}\;\mathrm{of}\;\mathrm{sample}\;\left(\mathrm g\right) \end{aligned}$$

The experiment was performed with three biological replicates, each contained three technical replicates.

Furthermore, the detection of lignin monomers in *SgPAL1* and *SgPAL2* OE lines was performed by Shanghai zcibio technology co, LTD. Briefly, Vanillin, and Syringaldehyde were purchased from Shanghai Yuanye Bio-Technology Co., Ltd (China) and prepared as 1.0 mg/mL standard stock solution of G, and S monomer, respectively. A 0.1 g sample powder was treated with NaOH hydrolysis, HCl neutralization, and ethyl acetate extraction. The organic phase was dried by nitrogen and dissolved in methanol and subsequently analyzed using Gas Chromatography-Mass Spectrometry (GC2010-QP2010, Sgimadzu Co., LTD, Japan) with an Agilrnt DB-5MS (30 m * 0.250 mm). The experiment was conducted with three biological replicates.

### Statisticla analysis

All data were calculated using Microsoft Excel 2019 (Microsoft Corporation, Redmond, WA, USA) and analyzed using GraphPad Prism 8.0.2 (GraphPad Software, La Jolla, CA, USA). The five indicators of disease in stylo were analyzed by one-way ANOVA, with different letter markers indicating significant differences (*P* < 0.05). The ANOVA-LSD test and t-test in IBM SPSS version 20 (Chicago, USA) were used to analyze statistical differences between materials or between treatments. *P < 0.05, **P < 0.01.

## Results

### Anthracnose resistance evaluation in *S. guianensis* germplasm

**Fig. 1:**
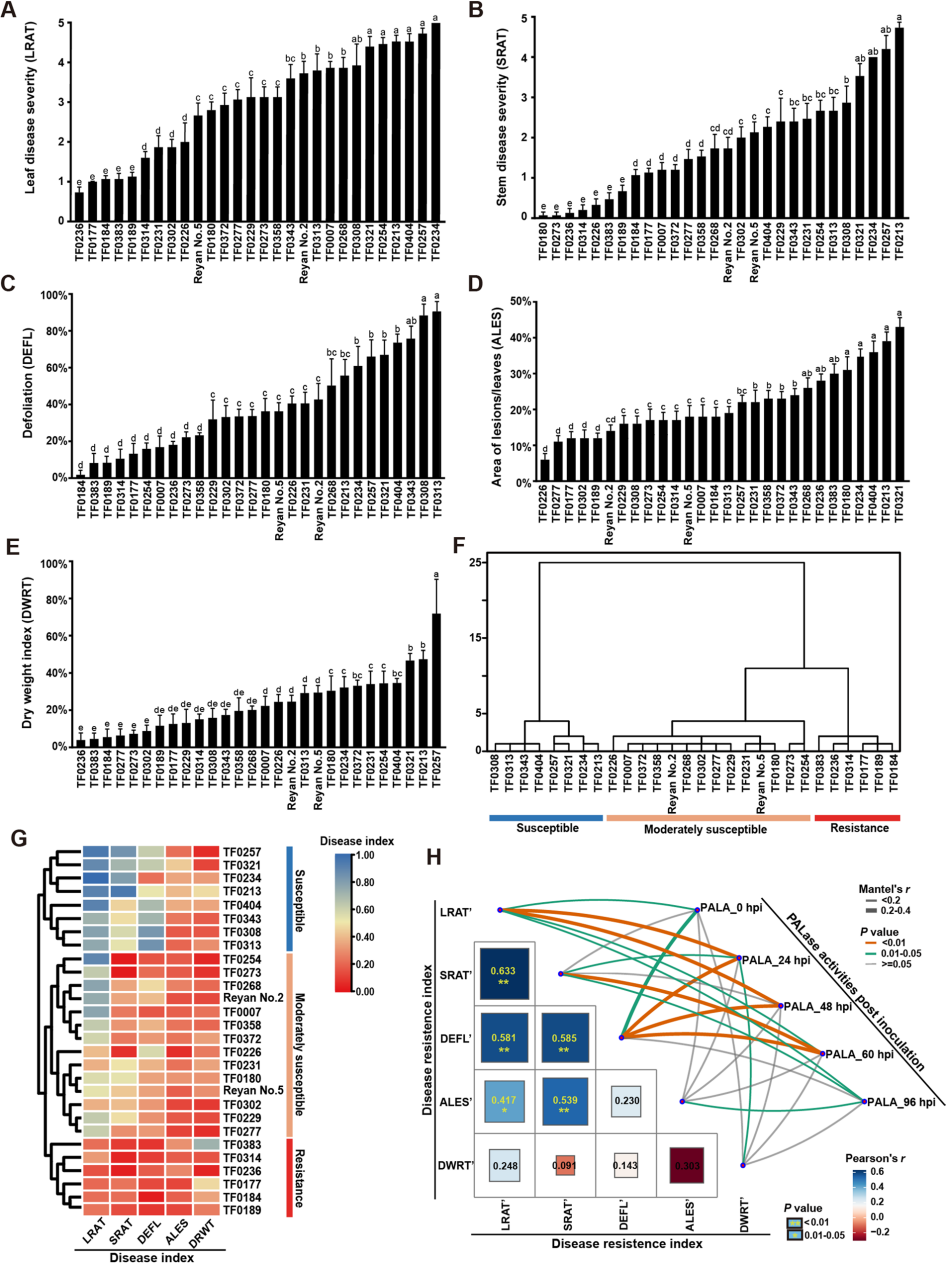
Evaluation of the resistance to *Colletotrichum gloeosporioides* DZ-19 in the core collection. The 28 accessions were spray-inoculated with spore suspension, and leaf disease severity rating (**A**), stem disease severity rating (**B**), defoliation (**C**), area of lesions/leaves (**D**) and dry weight index (**E**) were assessed. Different letters denote significant difference (*P* < 0.05). Clustering analysis of *S. guianensis* sensitivity (disease index) to DZ-19 were analyzed by SPSS (**F**) and TBtools (**G**). **H** Correlation analysis between the PALase activity and the five disease resistance indexes (LRAT’, SRAT’, DEFL’, ALES’, DWRT’) in the core collection after inoculation with DZ-19

To evaluate anthracnose resistance in the *S. guianensis*, 4-week-old plants of all 28 accessions were inoculated with *C. gloeosporioides* strain DZ-19 via spore suspension spraying. Disease assessment included multiple parameters: leaf (LRAT) and stem (SRAT) lesion severity and defoliation rate (DEFL) at 4 days post-inoculation (dpi), followed by lesion-to-leaf area ratio (ALES) at 6 dpi and dry weight reduction (DWRT) at 7 dpi. All five disease indicators exhibited continuous phenotypic variation across the accessions (Fig. 1A-E), supporting the quantitative nature of anthracnose resistance in stylo. Consistent results from Ward’s hierarchical clustering analysis using both SPSS and TBtools classified the accessions into three distinct groups: resistant (6 accessions), moderately susceptible (14 accessions), and susceptible (8 accessions) (Fig. 1F and G). This clear segregation of resistance phenotypes, coupled with the observed quantitative variation, establishes the collection as a valuable resource for identifying key genetic factors underlying anthracnose resistance.

### PAL activity correlates with anthracnose resistance in Stylo

Building on the known function of phenylalanine ammonia-lyase (PAL) in plant immunity, we assessed its enzymatic activity in 28 stylo accessions inoculated with *C. gloeosporioides*. Our analysis revealed significant positive correlations between PAL activity (60 hpi) and the disease resistance indices LRAT′, SRAT′, and DEFL′, with respective coefficients of 0.16, 0.25, and 0.34 (Fig. 1H). Furthermore, these three resistance parameters showed strong mutual correlations, all exceeding 0.581. Overall, the observed link between increased PAL activity and enhanced anthracnose resistance in the core collection implies that PAL-driven phenylpropanoid biosynthesis plays a functional role in conferring defense against *C. gloeosporioides* in stylo.

### Characterization and expression analysis of *SgPAL* gene family in response to *C. gloeosporioides* infection

**Fig. 2:**
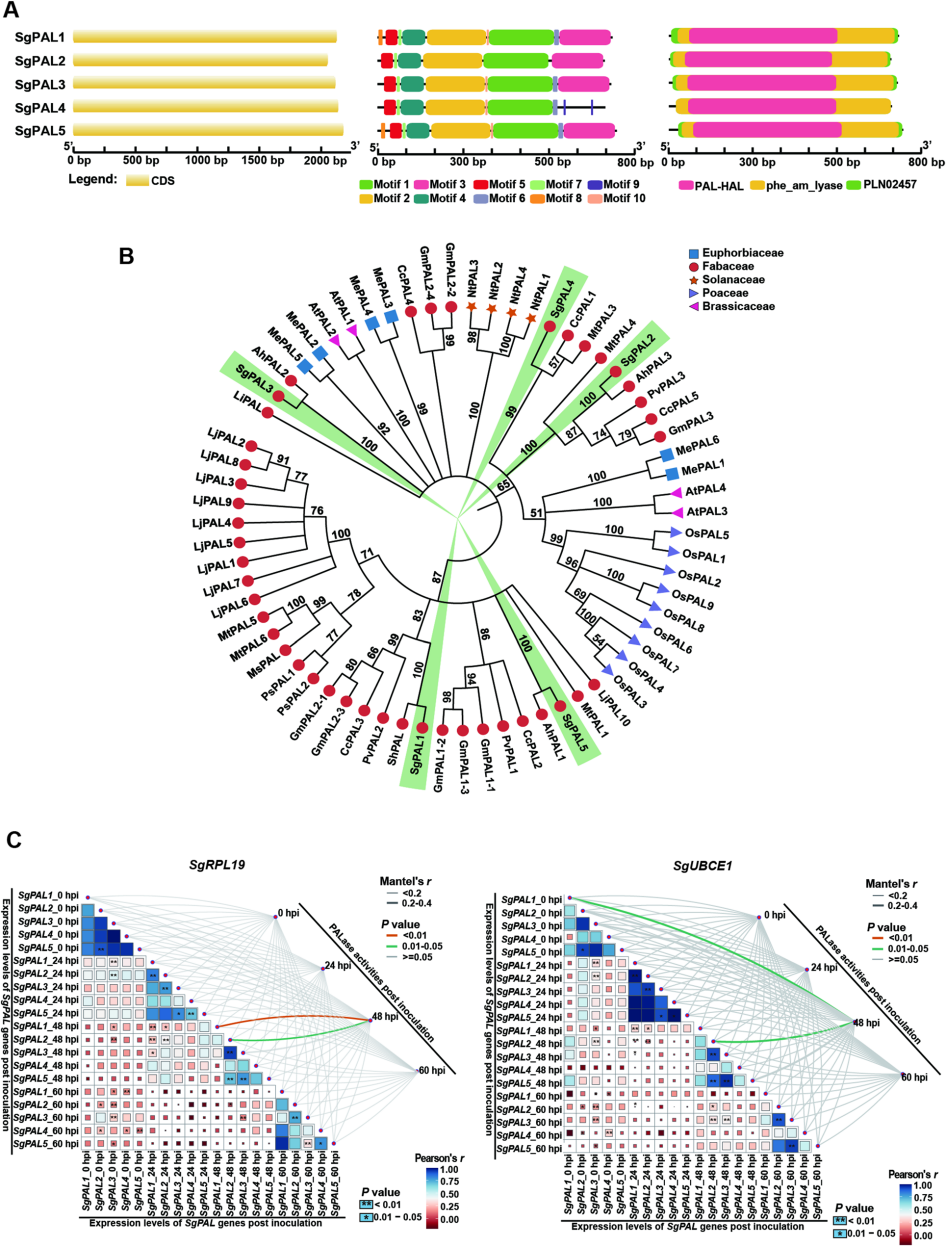
Sequence analysis of SgPALs and correlation analysis of gene expression levels with PALase activity in the core collection post inoculation. **A **The cds structures (left), sequence motifs (medium) and protein domains (right) of five members of the SgPAL gene family. **B **Phylogenetic analysis of SgPALs and 63 reported plants PAL proteins from five families. The red circles mark 45 PAL proteins derived from Fabaceae (*Stylosanthes guianensis = Sg*, *Stylosanthes humilis = Sh*, *Arachis hypogaea = Ah*, *Glycine max = Gm*, *Medicago truncatula = Mt*, *Medicago sativa = Ms*, *Phaseolus vulgaris = Pv*, *Cajanus cajan = Cc*, *Pisum sativum = Ps*, *Lotus japonicus = Lj*, *Leucaena leucocephala = Li*). The blue squares represent six PAL proteins from Euphorbiaceae (*Manihot esculenta = Me*). The rosy red triangles indicate the four Brassicaceae (*Arabidopsis thaliana = At*) PAL proteins. The yellow stars represent 4 PAL Solanaceae (*Nicotiana tabacum = Nt*). The purple triangles refer to nine PAL proteins from the Poaceae (*Oryza sativa = Os*). **C **Correlation analysis between internal leaf PALase activity and relative expression levels of five *SgPAL* genes in leaves of the core collection post inoculation of DZ-19. One-month-old plants were spray-inoculated with DZ-19. The PALase activity in leaves was assessed and the expression level of *SgPAL* genes were analyzed by qRT-PCR using the *SgUBCE1* and *SgRPL19* as internal reference genes

Transcriptome analysis of *C. gloeosporioides*-infected *S. guianensis* previously identified five PAL family genes (*SgPAL1-5*) that exhibited infection-responsive expression patterns. Bioinformatic characterization revealed these genes encode functionally intact phenylalanine ammonia-lyases, with CDS lengths ranging from 2076 to 2202 bp corresponding to 691–733 amino acid proteins. The predicted enzymes (75.6–80.3 kDa) shared characteristic features of plant PALs, including three conserved domains (PAL-HAL, pheam_lyase, and PLN02457) and the essential catalytic Ala-Ser-Gly motif (Table 1; Fig. 2A). Phylogenetic analysis demonstrated close evolutionary relationships between SgPALs and their orthologs in related *Fabaceae* species (*S. humilis*, *A. hypogaea*, and *C. cajan*), suggesting conserved functions in phenylpropanoid metabolism among these legumes (Fig. 2B).

**Table 1:** Sequence information of the PAL family members and characteristics of their proteins in *S. guianensis*

Gene name	Gene ID (NCBI)	length of coding DNA sequence (CDS)	Number of amino acids	Isoelectric point (pI)	Molecular weight (kDa)	Sublocation (BUSCA)	presence of conserved active site motif: Ala-Ser-Gly
*SgPAL1*	PQ788608	2148	715	6.14	77.86	cytoplasm	yes
*SgPAL2*	PQ788609	2076	691	5.82	75.84	cytoplasm	yes
*SgPAL3*	PQ788610	2136	711	6.06	77.73	cytoplasm	yes
*SgPAL4*	PQ788611	2160	719	5.94	78.83	cytoplasm	yes
*SgPAL5*	PQ788612	2202	733	6.09	79.74	cytoplasm	yes

The expression profiles of the *SgPAL* family during infection were quantified and normalized using two validated reference genes (*SgRPL19* and *SgUBCE1*). In the anthracnose-resistant cultivar ‘Reyan No.5’, the transcript levels of *SgPAL1*, *SgPAL2*, and *SgPAL3* were significantly up-regulated following inoculation (Fig. S1). In contrast, *SgPAL4* expression was suppressed, and *SgPAL5* showed no significant change. These distinct expression patterns suggest functional redundancy or divergence within the *SgPAL* gene family. Statistical analysis revealed a significant positive correlation between PAL enzyme activity at 48 hpi and the expression levels of both *SgPAL1* and *SgPAL2* at 48 hpi when normalized to the reference gene *SgRPL19* (Fig. 2C). Similarly, when *SgUBCE1* was used as the reference gene, PAL activity at 48 hpi showed significant correlations with *SgPAL1* expression at baseline (0 hpi) and *SgPAL2* expression at 48 hpi (Fig. 2C). These results implicate *SgPAL1* and *SgPAL2* as the primary mediators of defense-induced phenylpropanoid metabolism during *C. gloeosporioides* infection in *S. guianensis*.

### Biochemical and expression profiling of *SgPAL1/SgPAL2* in stylo defense

**Fig. 3:**
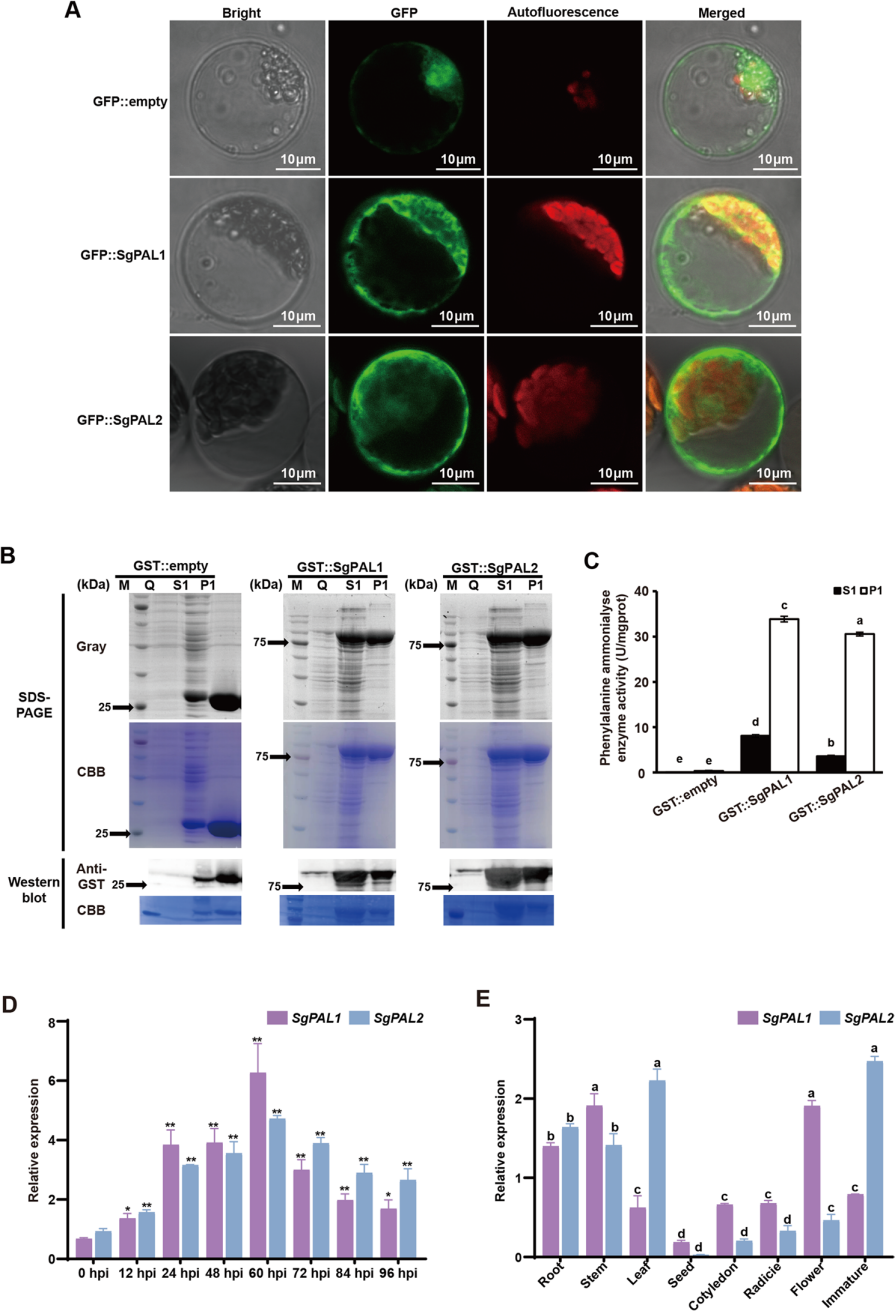
Subcellular Localization, biochemical, and expression pattern analysis of *SgPAL1* and *SgPAL2*. **A **Subcellular localization analysis of SgPAL1/2 using stylo protoplasts. The green fluorescence under confocal microscope represents the location of GFP protein or SgPAL-GFP fusion protein. Bars in each image represent the scale of 10 μm. Bright, bright filed; GFP, signal of green fluorescence; Autofluorescence, chloroplast autofluorescence; Merge, overlapping of Bright, GFP and autofluorescence. **B **Expression and purification of SgPAL1 and SgPAL2 proteins. **C **PALase activity assay of proteins before and after purification. The data are means of the enzyme activities determined from three independent plants ± SE. Different letters indicate significant difference in different groupings at *P < 0.05 by ANOVA-LSD test. M, 180 kDa protein Marker; Q, *E. coli* before IPTG induction; S1, *E. coli* after IPTG induction; P1, the GST::SgPAL fusion protein as purified by GST-Tag magnetic beads. **D **Expression analysis of *SgPAL1* and *SgPAL2* genes in stylo Reyan No.5 leaves in response to *C.gloeosporides* DZ-19 treatment. Data are the mean ± SE of four independent biological replicates (*n* = 4), Asterisks indicate significant difference in gene expression compared to 0 hpi by t-test, *P < 0.05, **P < 0.01. **E **Tissue-specific expression of *SgPAL1* and *SgPAL2* genes in stylo Reyan No.5. Data are the mean ± SE of three independent biological replicates (*n* = 3), different letters indicate significantly difference between groupings by Tukey’s multiple range test at *P* ≤ 0.05

To functionally characterize *SgPAL1* and *SgPAL2*, we isolated these genes from the anthracnose-resistant cultivar ‘Reyan No.5’ and conducted comprehensive analyses including subcellular localization, enzymatic properties, tissue-specific expression profiles, and pathogen-responsive regulation. Confocal microscopy revealed cytoplasmic localization of both SgPAL1 and SgPAL2 (Fig. 3A and Fig. S2), consistent with their predicted roles in phenylpropanoid metabolism.

For biochemical characterization, we successfully expressed and purified GST-tagged recombinant proteins in *E. coli* (Fig. 3B). Enzymatic assays demonstrated that both SgPAL1 and SgPAL2 exhibited significantly higher specific activity in the purified fractions compared to crude extracts when using L-phenylalanine as substrate (Fig. 3C), confirming their functional integrity as phenylalanine ammonia-lyases.

Expression profiling showed dynamic temporal regulation of both genes following pathogen challenge, with transcript levels peaking at 60 h post-inoculation (Fig. 3D). Spatial expression analysis revealed predominant accumulation in stems and leaves (Fig. 3E), the primary sites of *C. gloeosporioides* infection, suggesting their physiological relevance in defense responses.

### Functional validation of *SgPAL1/SgPAL2* in anthracnose resistance

**Fig. 4:**
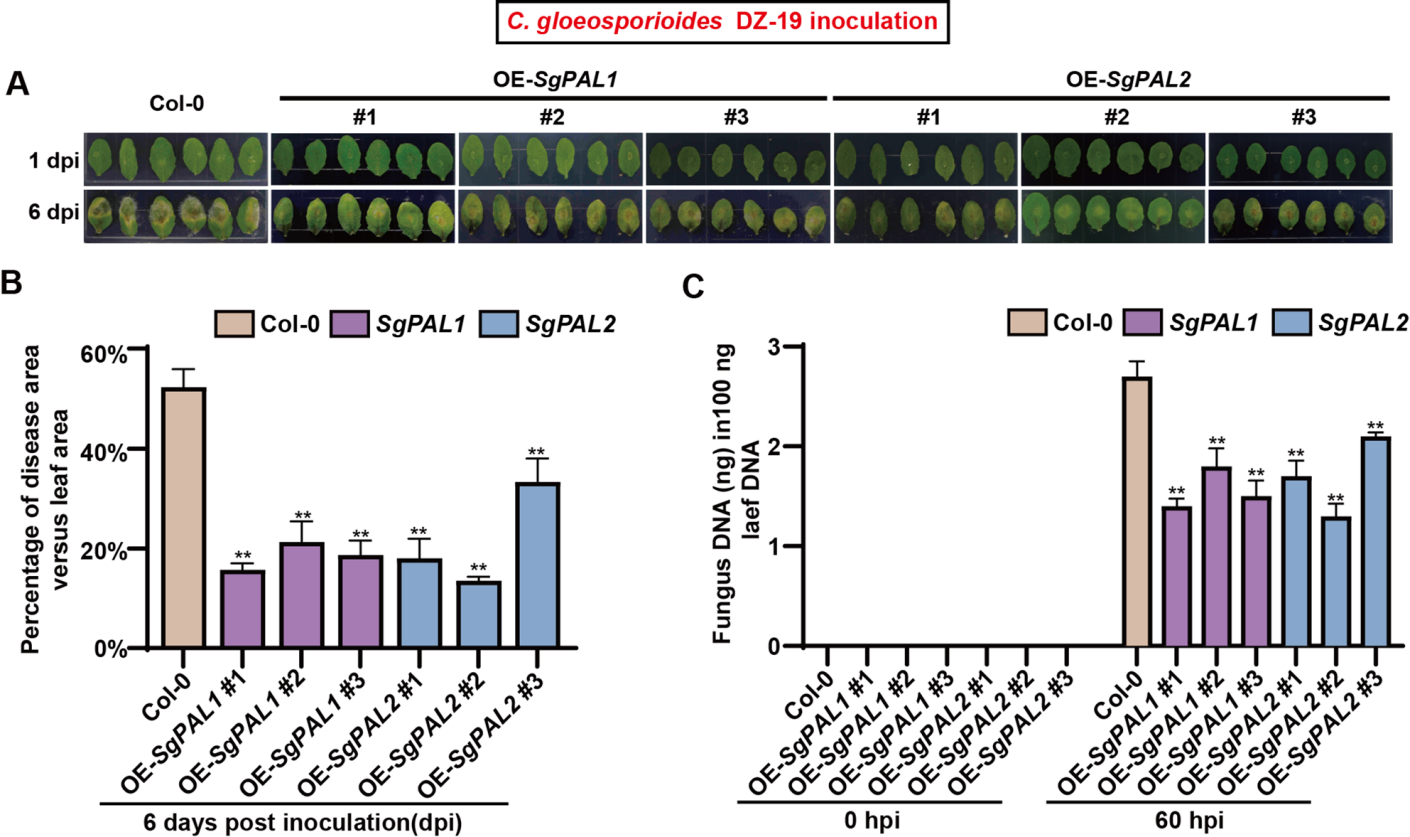
Pathogen assay on wild type (Col-0), *SgPAL1* and *SgPAL2* overexpression plants after inoculation with DZ-19. **A** Disease symptoms on leaves of Col-0, *SgPAL1* and *SgPAL2* overexpression plants on day 1 and day 6 post inoculation. **B **Mean percentage area of lesions on day 6 after drop inoculation on detached leaves. Data are the mean ± SE pooling from 12 leaves (*n* = 12). The experiment was repeated three times with similar results. Asterisks indicate a significant difference in overexpression lines compared to Col-0, according to a two-tailed t test using SPSS v. 20. *P < 0.05, **P < 0.01. **C **The contents of fungal DNA in 100 ng leaves of Col-0, *SgPAL1* and *SgPAL2* overexpression lines at 0 h and 60 h post-inoculation. Data are the mean ± SE pooling from 3 leaves (*n* = 6). Asterisks indicate significant difference in overexpression lines compared to Col-0 by t-test, **P < 0.01

To elucidate the defensive role of *SgPAL1* and *SgPAL2* against *C. gloeosporioides*, we generated and characterized transgenic *Arabidopsis* lines overexpressing these genes (OE *SgPAL1/SgPAL2*), with successful transgene integration and protein expression confirmed by molecular analyses (Fig. S3). Under standard growth conditions, the *OE-SgPAL1* and *OE-SgPAL2* transgenic lines were morphologically indistinguishable from the wild-type controls and showed no signs of growth penalty or developmental delay. Pathogen assays demonstrated the enhancement of resistance in transgenic plants, with *SgPAL1*-OE lines showing 9–30% reduction in lesion area and 31–53% decrease in fungal biomass compared to wild-type (Col-0) controls. The protective effect was also shown in *SgPAL2*-OE lines, exhibiting 5–35% smaller lesions and 22–51% lower fungal colonization (Fig. 4 and Fig. S4).

**Fig. 5:**
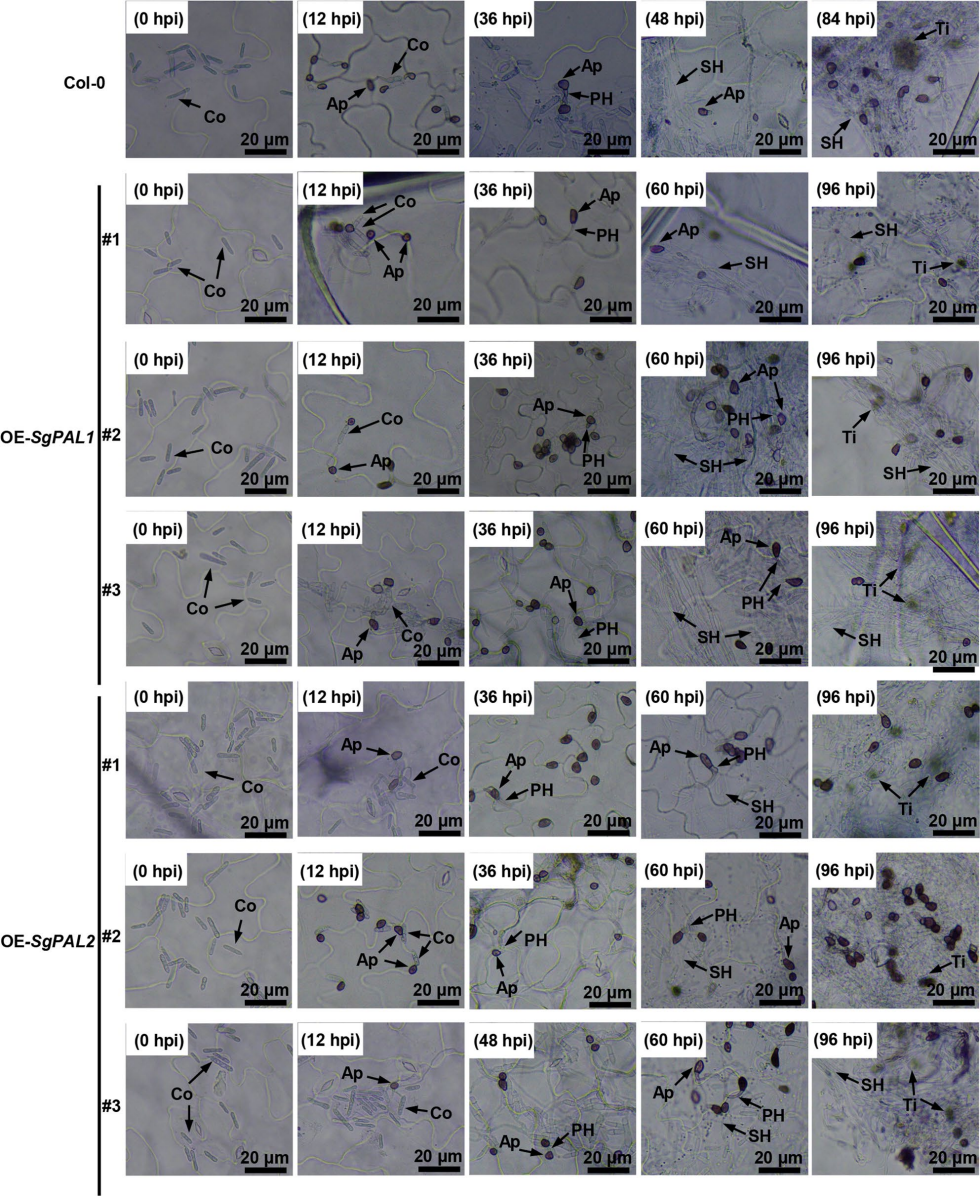
Microscopic observation of the development of *C. gloeosporioides* in Col-0, *SgPAL1* and *SgPAL2* overexpression plants. Conidia (Co) attach to the leaf at 0 h post inoculation (hpi). Appressoria (Ap) were observed at 12 hpi. The primary hyphae (PH) were observed at 36 hpi for Col-0, OE-*SgPAL1* (#1, #2, and #3) and OE-*SgPAL2* (#1 and #2), which was captured at 48 hpi in OE-*SgPAL2* #3. The PH develop into secondary hyphae (SH) at 48 hpi for Col-0, and at 60 hpi for OE-*SgPAL1* and OE-*SgPAL2*. The brown tissue liquid (Ti) flows out at 84 hpi for Col-0, and at 96 hpi for OE-*SgPAL1* and OE-*SgPAL2*. Bars = 20 μm

Microscopic examination of infection dynamics revealed that both transgenes significantly delayed fungal pathogenesis. The developmental progression of *C. gloeosporioides* was retarded by 12–24 h in transgenic plants, with secondary hyphae emerging at 60 hpi (vs. 48 hpi in WT) and completing the infection cycle by 96 hpi (vs. 84 hpi in WT) (Fig. 5). These consistent observations across multiple parameters demonstrate that *SgPAL1* and *SgPAL2* overexpression confers enhanced and prolonged resistance against anthracnose in *Arabidopsis*.

In summary, the disease symptoms, pathogen assays, and infection process in OE *SgPAL1*/*SgPAL2* after the inoculation of *C. gloeosporioides* were comparable to those described in the established *Arabidopsis thaliana*-*Colletotrichum gloeosporioides* pathosystem, indicating that these transgenic lines represent effective materials for explore anthracnose resistance mechanisms [28, 29].

### Transcriptomic insights into *SgPAL*-mediated anthracnose resistance

To decipher the molecular mechanisms underlying *SgPAL*-conferred resistance, we performed comparative transcriptome analysis of wild-type (Col-0) and four transgenic lines (two OE-*SgPAL1* and two OE-*SgPAL2*) at pre-infection (0 hpi) and peak defense response (60 hpi) stages. Principal component analysis demonstrated clear separation between inoculated and non-inoculated samples, with high reproducibility among biological replicates (Fig. S5A). The transgenic lines exhibited extensive transcriptional reprogramming, displaying 9,457 − 13,661 differentially expressed genes (DEGs) compared to wild-type controls (Tables S4 and S5). Comparative analysis identified 759 and 563 unique DEGs specifically associated with *SgPAL1* and *SgPAL2* overexpression, respectively (Fig. S5B).

**Fig. 6:**
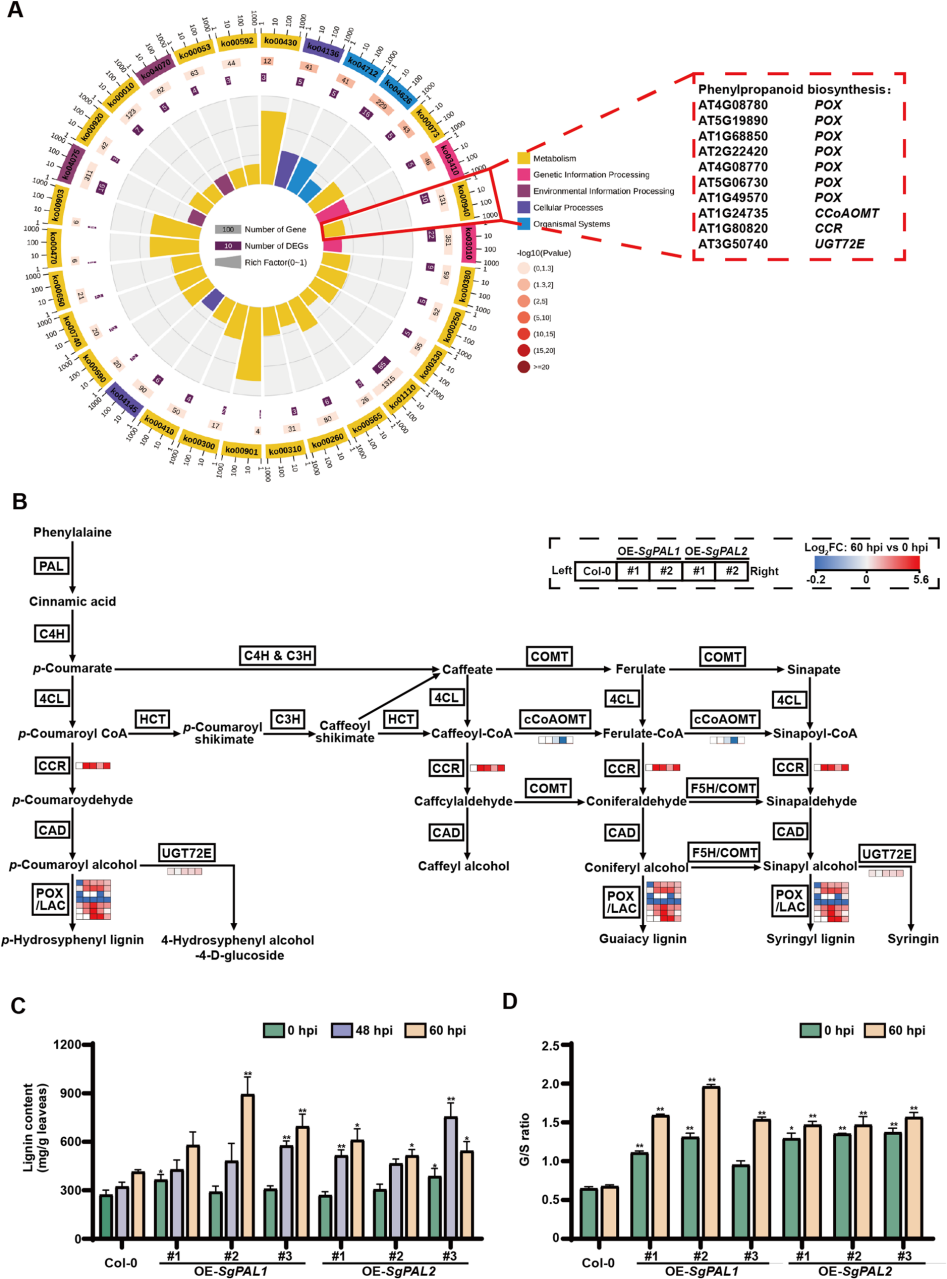
Transcriptomic analysis and detection of lignin content in Col-0, *SgPAL1* and *SgPAL2* overexpression plants after inoculation with *C. gloeosporioides* DZ-19. Transcriptional sequencing was conducted on leaves of Col-0, OE-*SgPAL1* (#1, #2), and OE-*SgPAL2* (#1, #2) *Arabidopsis* at 0 h and 60 h post-inoculation with DZ-19. **A **KEGG enrichment analysis of common DEGs in OE-*SgPAL1 or* OE-*SgPAL2* compared to Col-0. Among them, 10 differential genes were detected in the phenylpropanoid biosynthesis pathway (k00940). **B **The transcriptional profiles of 10 DEGs involved in the phenylpropanoid biosynthesis in Col-0 and overexpression lines. Data represent the mean of three biological replicates. PAL, phenylalanine ammonia-lyase; C4H, cinnamate 4-hydroxylase; 4CL, 4-coumarate CoA ligase; C3H, p-coumarate 3 hydroxylase; HCT, hydroxycinnamoyl transferse; F5H, ferulate 5-hydroxylase; CCR, cinnamoyl CoA reductase; CAD, cinnamyl alcohol dehydrogenase; COMT, caffeic acid 3-O-methyltransferase; CCoAOMT, caffeoyl-CoA O-methyltransferase; LAC, laccase; POX, peroxidase. Log_2_FC, Log_2_Fold Change. **C **The contents of lignin in leaves of Col-0, *SgPAL1* and *SgPAL2* overexpression plants at 0 h, 48 h and 60 h post-incolution with DZ-19. **D **The ratio of lignin G to S monomer in leaves at 0 hpi and 60 hpi. Data are the mean ± SE pooling from three biological replicates (*n* = 3). Asterisks indicate significant difference in overexpression lines compared to Col-0 by t-test, *P < 0.05, **P < 0.01

A core set of 10 phenylpropanoid pathway genes (ko00940) showed consistent regulation in both transgenic lines (Fig. 6A). Notably, *CCR* (*At1g80820*) and multiple peroxidase genes (*At4g08780*, *At5g19890*, *At4g08770*, *At5g06730*, *At1g49570*) exhibited significantly stronger induction in transgenic plants compared to wild-type. In contrast, cCoAoMT (*At1g24735*), involved in S-unit synthesis, showed modest downregulation in selected transgenic lines. The monolignol glucosyltransferase UGT72E (*At3G50740*) displayed marked upregulation across all genotypes following infection (Fig. 6B). These coordinated transcriptional changes indicate that *SgPAL*-mediated resistance involves substantial remodeling of the phenylpropanoid pathway, likely leading to altered lignin deposition patterns that enhance cell wall fortification against *C. gloeosporioides* invasion.

To further explore the molecular mechanisms beyond lignin deposition, we investigated the expression of salicylic acid (SA) biosynthesis genes given that PAL is upstream of SA biosynthesis. Notably, two key SA biosynthetic genes, *ICS1* (*At1g74710*) and *PBS3* (*At5g13320*), were consistently up-regulated in both transgenic lines compared to the wild-type plants (Fig. S6). This concerted induction suggests that *SgPAL1/SgPAL2*-mediated resistance is not solely dependent on cell wall reinforcement but also involves the activation of the SA signaling pathway, which likely contributes to the enhanced defense against *C. gloeosporioides*.

### Lignin metabolism contributes to *SgPAL*-mediated anthracnose resistance

To elucidate the role of lignin in *SgPAL*-conferred resistance, we conducted comprehensive analysis of lignin accumulation and composition during *C. gloeosporioides* infection. Pathogen challenge induced significant lignin deposition in both wild-type (Col-0) and transgenic plants, with *SgPAL*-overexpressing lines consistently showing enhanced lignin accumulation compared to controls (Fig. 6C). Notably, this lignification response was accompanied by a marked shift in monomeric composition, as evidenced by the significantly higher guaiacyl-to-syringyl (G/S) ratio observed in transgenic lines relative to wild-type plants following infection (Fig. 6D and Fig. S7). These findings position SgPAL1/2 as key regulators of both the total lignin and the G/S ratio during plant defense responses.

## Discussion

*Colletotrichum* spp. are devastating fungal pathogens responsible for anthracnose in a wide range of crops, including legume forages [30]. Their high genetic variability limits the effectiveness of conventional control strategies, such as physical isolation and chemical treatments [6]. In this study, we clearly classified the resistance phenotypes of 28 core accessions of *S. guianensis*, a tropical diploid forage legume, against *C. gloeosporioides*. Notably, six accessions demonstrated significantly stronger resistance than the commercial cultivars “Reyan No.2” and “Reyan No.5” (Fig. 1) [31], underscoring their potential use in breeding programs. Furthermore, our findings indicate that anthracnose resistance in *Stylosanthes* involves not only an increase in total lignin content, but more critically, a qualitative shift in lignin monomer composition. This insight represents a significant advance in elucidating the molecular basis of disease resistance in forage legumes.

The 28 *S. guianensis* proved to be an effective platform for dissecting anthracnose resistance mechanisms. Our hierarchical correlation analyses revealed that pathogen-induced PAL activity positively correlated with resistance indices (LRAT’ and SRAT’) (Fig. 1). PAL, a member of the ammonia-lyase superfamily, often exhibits functional redundancy and differentiation among its gene family members. In the resistant cultivar Reyan No.5, infection by *C. gloeosporioides* transiently up-regulated the expression of *SgPAL1*, *SgPAL2*, and *SgPAL3*, with peak levels observed at 48–60 h post-inoculation (Figure S1). This induction pattern parallels the response of *AhPAL1* and *AhPAL2* in peanut to *Aspergillus flavus* infection [33]. Conversely, the expression of *SgPAL4* and *SgPAL5* was either significantly suppressed or remained unchanged (Figure S1), a trend similar to that of *AhPAL5* and *AhPAL6*, suggesting their primary functions may lie outside of disease resistance [33]. These collective findings indicate that *SgPAL1*, *SgPAL2*, and *SgPAL3* play a cooperative role in the defense response against anthracnose. Furthermore, the expression levels of *SgPAL1* and *SgPAL2* were strongly associated with PAL enzymatic activity in 28 stylo, suggesting their regulatory role in disease resistance (Fig. 2C). Owing to the current absence of an efficient genetic transformation system in stylo, we conducted functional validation of *SgPAL1* and *SgPAL2* using an *Arabidopsis thaliana*-*C. gloeosporioides* pathosystem. This alternative experimental system was selected based on the pathogen’s ability to infect both stylo and *Arabidopsis* with comparable virulence, thereby enabling reliable cross-species functional analysis of these candidate resistance genes [8, 33]. *Arabidopsis* overexpression (OE) lines confirmed that *SgPAL1* and *SgPAL2* enhance resistance to anthracnose, consistent with previous findings in other pathosystems, including rice–*Magnaporthe oryzae* [32], soybean–*Phytophthora sojae* [13], and peanut–*Aspergillus flavus* [33]. This functional conservation across diverse plant-pathogen interactions underscores the potential for cross-species application of PAL-mediated resistance mechanisms. Given its diploid and self-pollinating nature, *S. guianensis* serves as an excellent genetic model for studying anthracnose resistance. The resistance-associated genes identified here, particularly *SgPAL1* and *SgPAL2*, may provide valuable insights for improving disease tolerance in more genetically complex forage crops, such as the tetraploid *M. sativa* (alfalfa) and *S. cannabina* (sesbania). It should be noted that our functional validation was performed in a heterologous system (*Arabidopsis thaliana*). While this approach convincingly demonstrates the sufficiency of *SgPAL1/2* in conferring resistance, it does not establish their necessity in the native *Stylosanthes* host, owing to the absence of an efficient genetic transformation system for this species. To unequivocally confirm the essential role of *SgPAL1/2* in *Stylosanthes-Colletotrichum* interaction, future work should focus on establishing a stable genetic transformation and CRISPR/Cas9-mediated gene editing platform for *Stylosanthe*s. Targeted knockout of *SgPAL1/2* would allow for a direct assessment of their necessity in disease resistance within the native host. Moreover, the cross-species validation will also be essential to assess the translational potential of our findings.

Plant phenylalanine ammonia-lyase (PAL) plays a pivotal role in disease resistance by regulating downstream metabolites of the phenylpropanoid pathway [11, 34]. As a key product of this pathway, lignin serves as a structural component of plant cell walls and contributes to pathogen defense through both physical and biochemical mechanisms [35, 36]. Our functional characterization of *SgPAL1/2* in stylo demonstrates its critical role in anthracnose resistance by modulating lignin biosynthesis at two distinct levels: quantitatively increasing total lignin deposition and qualitatively altering the guaiacyl (G) to syringyl (S) ratio (Fig. 6 and Fig. S6). Lignin acts as a primary physical barrier against pathogen invasion. Upon infection, localized lignin deposition reinforces cell walls at penetration sites, forming a structural defense that impedes fungal hyphal growth and enzymatic degradation [37]. This lignification response has been documented across diverse plant species, including *Arabidopsis thaliana*, tobacco (*Nicotiana tabacum*), rice (*Oryza sativa*), and poplar (*Populus* spp.), where pathogen challenge induces lignin biosynthetic genes and enhances cell wall fortification [10, 38, 39]. Beyond its structural role, lignin composition significantly influences defense efficacy. Comparative studies indicate that lignin derived from p-coumaryl (H) and coniferyl (G) alcohols exhibits stronger antifungal activity against white-rot fungi such as *Trametes versicolor* and *Rhodonia placenta* than lignin derived from sinapyl (S) alcohol [40]. These enhanced antimicrobial properties are associated with specific structural features in G/H-lignin subunits, such as carbonyl groups and conjugated double bonds, which are more effective at inhibiting fungal growth [40, 41]. Hemibiotrophic pathogens such as *Colletotrichum gloeosporioides*, *Verticillium dahliae*, and *Magnaporthe oryzae* share a common infection strategy involving host cell penetration, intracellular expansion, and cellulase secretion [42, 43]. For instance, overexpression of *GhLAC15* enhances cell wall lignification, leading to increased total lignin, G monolignol content, and G/S ratio, which significantly improves *Verticillium* wilt resistance in transgenic *Arabidopsis* [43]. Similarly, in rice, elevated lignin content and an increased G/S ratio result in pronounced thickening of sclerenchyma cells near the epidermis, inhibiting *Magnaporthe oryzae* penetration during early infection [36]. The predominance of G-lignin may confer resistance not only by reinforcing cell wall integrity through its highly cross-linked structure but also by directly interfering with pathogen-secreted cell wall-degrading enzymes during the necrotrophic phase [43, 44]. It can be inferred that increased lignin content and G/S ratio contribute to resistance by thickening the cell wall, thereby hindering *C. gloeosporioides* infection. Our findings are consistent with these established mechanisms, as *SgPAL1/2* overexpression elevated both total lignin content and the G/S ratio, a compositional shift correlated with enhanced resistance to *C. gloeosporioides*. While enhanced lignification improves resistance in forage crops like stylo, it may also reduce digestibility. Therefore, a balance between defense and forage quality must be considered in breeding applications. We propose an inducible modification strategy in which pathogen-responsive promoters drive lignin biosynthesis genes exclusively during infection. Such targeted regulation could maintain baseline digestibility while activating defense-related lignification when needed, potentially achieving an optimal balance between these competing traits. Future research should explore tissue-specific or temporally controlled expression systems to implement this strategy. For the field of forage legume biology, our discovery that disease resistance involves reprogramming of lignin biosynthesis offers a new breeding target. Rather than selecting solely for overall lignin content, future efforts could focus on fine-tuning lignin composition to enhance durable resistance without compromising forage quality.

Our transcriptomic analysis of *SgPAL1* and *SgPAL2* overexpression (OE) lines revealed a tightly coordinated gene network that simultaneously modulates total lignin content and G/S ratio through multiple synergistic mechanisms (Fig. 6). The upregulation of *CCR* and *PRX* genes, induction of *UGT72E*, and downregulation of *cCoAoMT* collectively drive the observed lignification patterns associated with enhanced disease resistance (Fig. 6). The metabolic shift begins with *CCR* upregulation, which serves as the critical control point for carbon flux into the phenylpropanoid pathway. As the first committed enzyme in monolignol biosynthesis, increased *CCR* activity directly elevates total lignin production, consistent with its established role in lignin accumulation across species [45]. This effect is further amplified by the concurrent downregulation of *cCoAoMT*, which reduces the metabolic flow toward S-lignin precursors [39]. Such coordinated regulation of these opposing enzymes creates a biochemical bottleneck that preferentially accumulates G-lignin precursors while maintaining high overall lignin output. The polymerization process is similarly fine-tuned through the induction of *PRX* genes and *UGT72E* [41]. Peroxidases and laccases encoded by *PRX* not only catalyze the oxidative coupling of monolignols but also exhibit intrinsic substrate specificity that favors G-unit incorporation into the growing lignin polymer [41, 46]. Meanwhile, UGT72E-mediated glucosylation provides spatial control over lignin deposition while differentially stabilizing G-monomers through selective glycosylation [47]. These post-synthetic modifications work in concert with the upstream metabolic changes to establish both the quantity and quality of lignin deposition. This integrated regulatory cascade explains how *SgPAL1/2* overexpression achieves dual control over lignin properties. By simultaneously activating CCR-driven biosynthesis, suppressing cCoAoMT-mediated S-precursor production, and modulating polymerization through PRX and UGT72E, the transgenic lines develop a lignin profile optimized for structural defense. These findings reveal a sophisticated, multi-layered regulatory network that coordinates metabolic flux, monomer selection, and polymer assembly to produce defense-adapted lignification patterns.

Salicylic acid (SA) is a pivotal plant defense hormone, essential for immunity against biotrophic and hemibiotrophic pathogens [48]. For instance, enhanced salicylic acid signaling improves resistance in rice to both *Xanthomonas oryzae pv. Oryzae* (biotrophic pathogen) and *Magnaporthe oryzae* (hemibiotrophic pathogens) [49]. Further studies have shown that in apple, salicylic acid signaling is triggered by *Colletotrichum gloeosporioides* (hemibiotrophic pathogens) infection and ultimately strengthens resistance to anthracnose through the promotion of lignin synthesis [50]. In plants, SA biosynthesis occurs primarily via two pathways: the isochorismate synthase (ICS) pathway, which is the major route in Arabidopsis, and the phenylalanine ammonia-lyase (PAL) pathway, which plays a more prominent role in certain species such as soybean, rice, maize, and tobacco [48]. Our transcriptome data further substantiate the involvement of SA signaling in *SgPAL1/2*-mediated resistance. Specifically, the consistent upregulation of key SA biosynthetic genes (e.g., *ICS1*, *PBS3*) in our overexpression lines (Fig. S6) suggests a potential positive feedback loop or crosstalk between the PAL and ICS pathways. However, direct evidence linking SA accumulation to resistance in stylo remains to be established. Future work should include direct measurement of SA levels in transgenic plants upon pathogen challenge and employ genetic approaches, such as disrupting SA signaling in the *SgPAL1/2* overexpression background, to conclusively determine the contribution of SA to the observed resistance phenotype.

Notably, *SgPAL1* and *SgPAL2* exhibit both functional redundancy and differentiation. While they share 71.2% sequence similarity and possess identical conserved active sites (Ala-Ser-Gly) and functional domains, indicative of functional conservation, pronounced differences emerged in their tissue-specific expression patterns. Despite being co-induced and peaking at 60 h post-inoculation with *C. gloeosporioides*, *SgPAL1* expression was significantly higher than *SgPAL2* in cotyledons, roots, and flowers, suggesting a potential secondary role for *SgPAL1* in plant development. Functional validation in an *Arabidopsis* overexpression system confirmed that both genes enhance resistance to *C. gloeosporioides* and promote lignin deposition, albeit to different extents. Following inoculation, the OE-SgPAL2 #3 line developed moderately larger lesions and accumulated higher fungal biomass than the OE-SgPAL1 lines. Furthermore, *SgPAL1* overexpression led to marginally greater increases in both total lignin content and the G/S ratio, highlighting a quantitative difference in the efficacy of their disease resistance functions. From a breeding perspective, our findings demonstrate that heterologous expression of either gene significantly increases disease resistance. A key question for future research is whether coordinated expression of both genes in the native host would yield additive or synergistic effects, potentially leading to more robust and durable resistance.

## Conclusion

In summary, systematic evaluation of anthracnose resistance phenotypes, PAL enzyme activities, and *SgPAL* gene expression patterns in 28 *S. guianensis* accessions following *C. gloeosporioides* inoculation identified *SgPAL1* and *SgPAL2* as pivotal isoforms governing both PAL activity variation and disease resistance in the germplasm. Functional characterization revealed that these genes confer anthracnose resistance by modulating the lignin biosynthesis pathway, evidenced by increased total lignin content and altered guaiacyl/syringyl (G/S) monomer ratios upon their induction, which likely enhances cell wall lignification to defend against the pathogen. Our findings provide valuable genetic resources for resistance breeding, while *SgPAL1* and SgPAL2 stand out as prime molecular targets for engineering anthracnose-resistant legume forages.

## Supplementary Information

Supplementary Material 1.

Supplementary Material 2.

Supplementary Material 3.

## Data Availability

All the data have been provided in the main manuscript and supplementary files. The raw Illumina reads generated from RNAseq experiments were deposited at NCBI SRA under the accession number: PRJNA1199688. Sequence data of *SgPAL* genes from this study can be found in the database of NCBI under the following accession numbers: PQ788608(*SgPAL1*), PQ788609(*SgPAL2*), PQ788610(*SgPAL3*), PQ788611(*SgPAL4*), PQ788612(*SgPAL5*).
