# Supplementary material for: SgPAL1/2 confers anthracnose resistance in Stylosanthes guianensis by modulating lignin content and monomer ratios
Source: BMC Plant Biol. 2025 Dec 24;25:1727. doi: 10.1186/s12870-025-07720-2 (PMC12729147; doi:10.1186/s12870-025-07720-2)
Supplement: Supplementary file 1 — Supplementary Material 1. [file 12870_2025_7720_MOESM1_ESM.pdf]

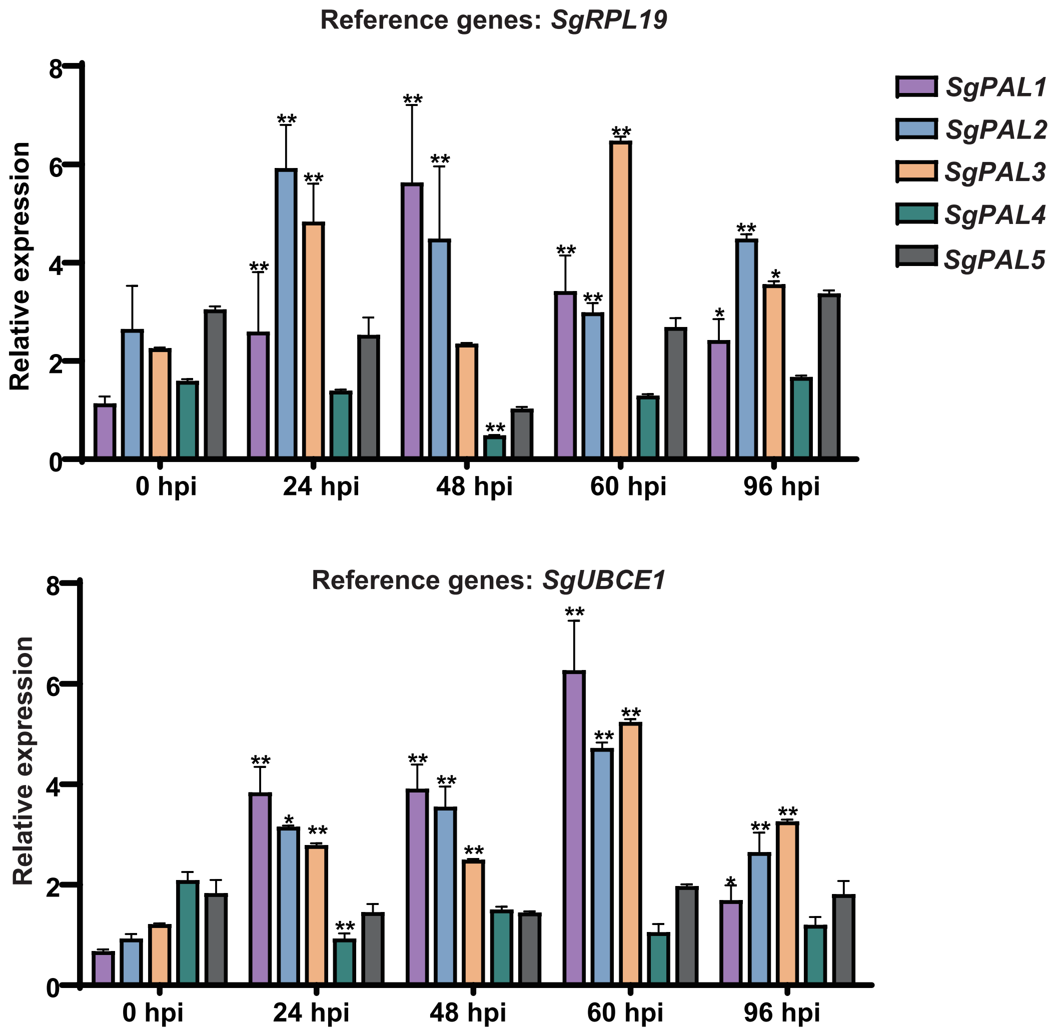

Fig. S1. Expression analysis of *SgPALs* gene family memebers in stylo Reyan No.5 leaves in response to *C.gloeosporides* DZ-19 treatment. Data are the mean ± SE of four independent biological replicates (n = 4), Asterisks indicate significant difference in gene expression using the *SgRPL19* and *SgUBCE1* as internal reference genes compared to 0 hpi by t-test, *P < 0.05, **P < 0.01.

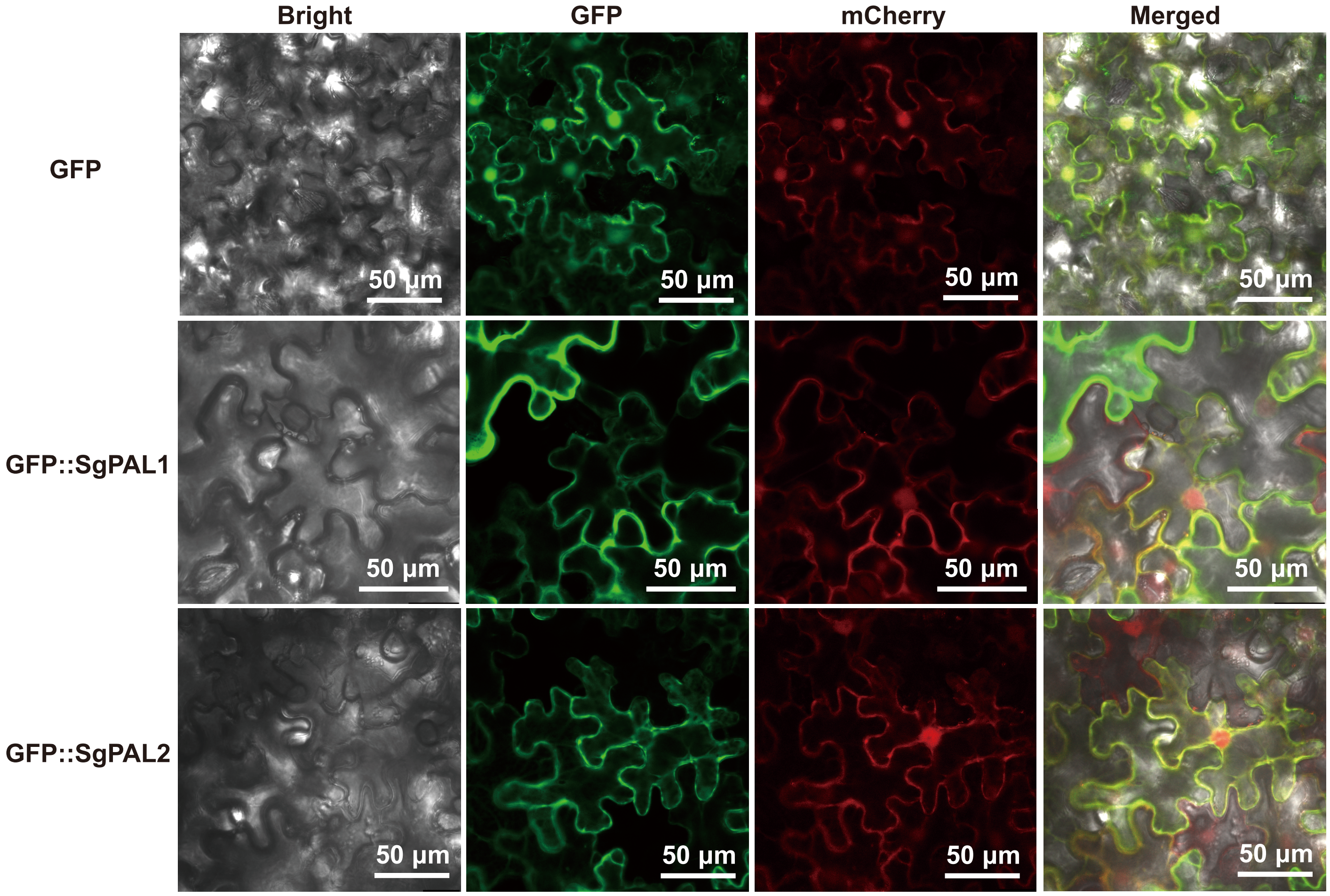

Fig. S2. Subcellular localization of SgPAL1 and SgPAL2 proteins in *Nicotiana benthamiana.* Co-localization of SgPAL1/2-GFP and mCherry protein. Images were taken using confocal microscopy 2 days after infiltration in *N. benthaminan*. Bright, bright filed; GFP, signal of green fluorescence; mCherry, marker for nucles and cytoplasm protein mCherry fluorescence; Merge, overlapping of Bright, GFP and mCherry fluorescence.

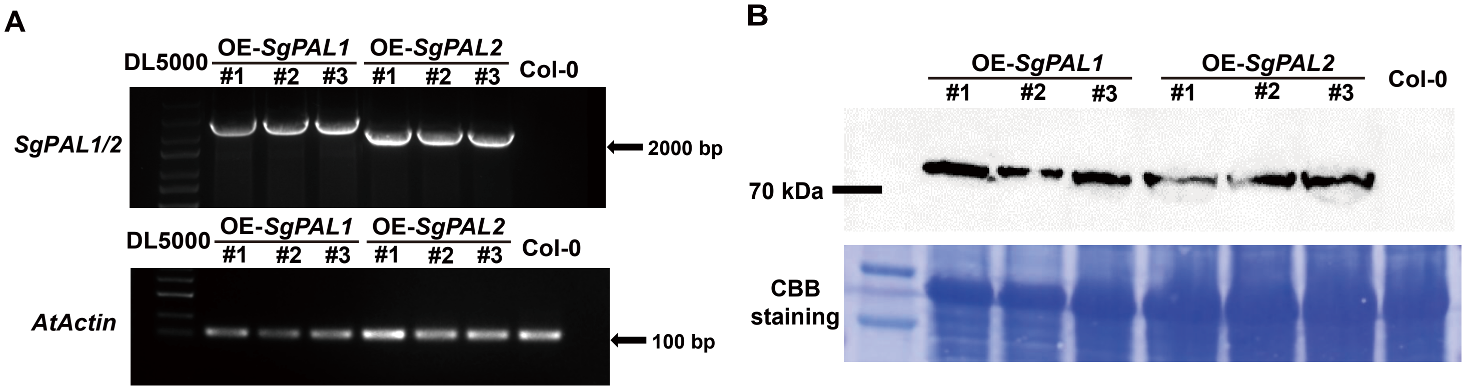

Fig. S3. Confirmation of *Arabidopsis SgPAL1* and *SgPAL2* overexpression lines. (A) RT-PCR detection of *SgPAL1*, *SgPAL2* or internal reference gene *AtActin*. (B) Weatern blot detection of SgPAL1-myc and SgPAL2-myc fusion proteins.

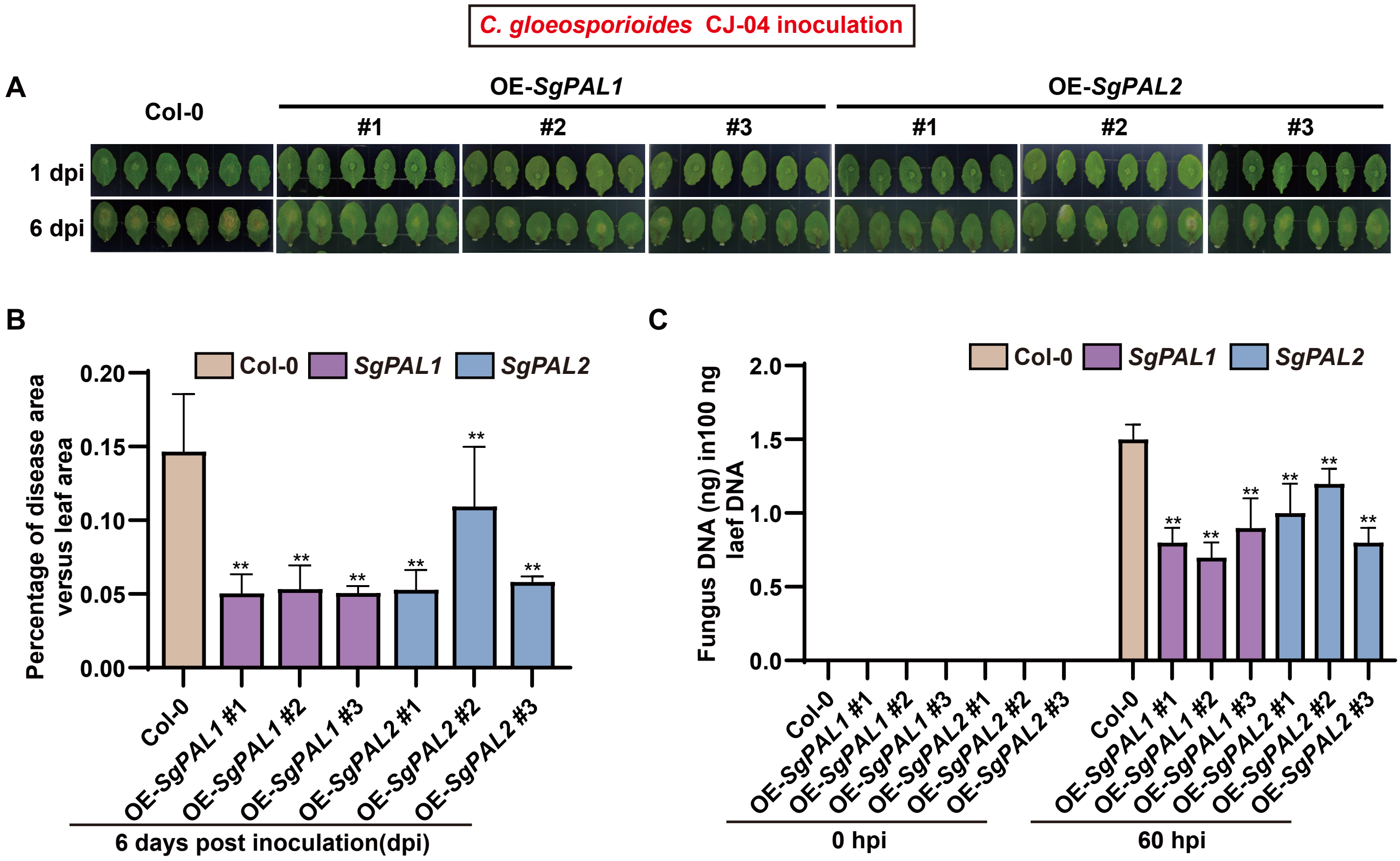

Fig. S4. Pathogen assay on wild type (Col-0), *SgPAL1* and *SgPAL2* overexpression plants with *C. gloeosporioides* CJ-04. (A) Symptoms of disease on detached leaves of Col-0, *SgPAL1* and *SgPAL2* overexpression lines on day 1 and day 6 after drop inoculation with spore suspension. (B) Mean percentage area of lesions on day 6 after drop inoculation on detached leaves. Data are the mean ± SE pooling from 12 leaves (n=12). The experiment was repeated three times with similar results. Asterisks indicate a significant difference in overexpression lines compared to Col-0, according to a two-tailed t test using SPSS v. 20. *P < 0.05, **P < 0.01. (C) The contents of fungal DNA in 100 ng leaves of Col-0, SgPAL1 and SgPAL2 overexpression lines at 0 h and 60 h post-incolution. Data are the mean ± SE pooling from 3 leaves (n=6). Asterisks indicate significant difference between in overexpression lines compared to Col-0 by t-test, **P < 0.01.

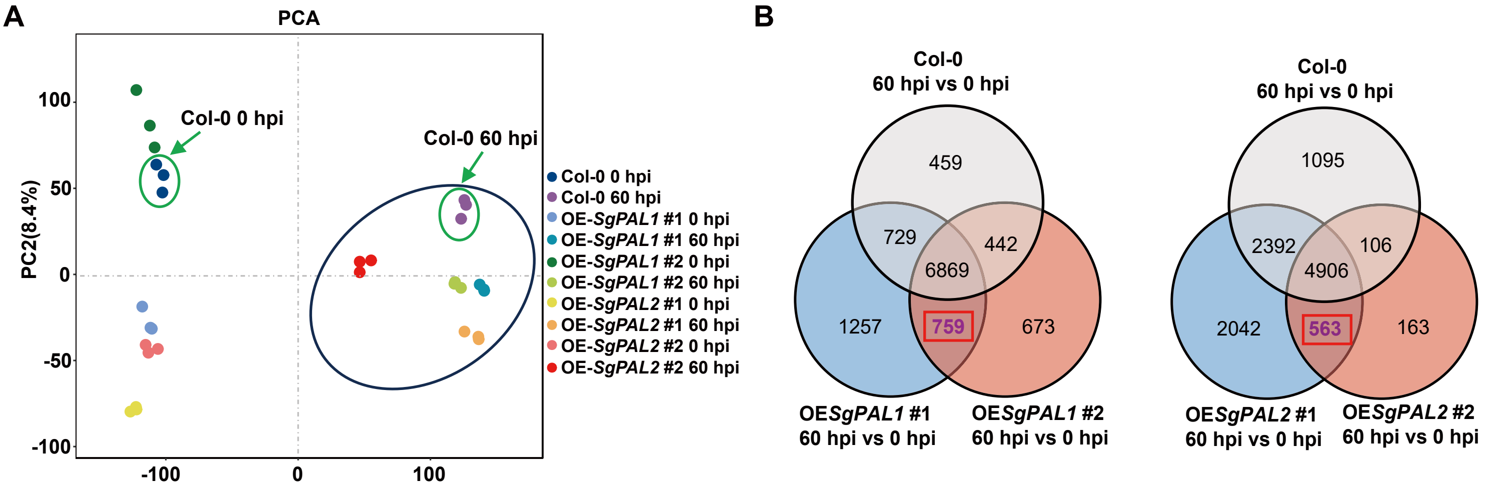

Fig. S5. Principal compontent (PCA) and venn diagram analysis of transcriptomic data of Col-0, *SgPAL1* and *SgPAL2* overexpression plants at 0 h and 60 h post inoculation. (A) PCA. (B) Venn diagram analysis of differentially expressed genes (DEGs) in response to *C. gloeosporioides* infection in Col-0, OE-*SgPAL1* (#1, #2) and OE-*SgPAL2* (#1, #2).

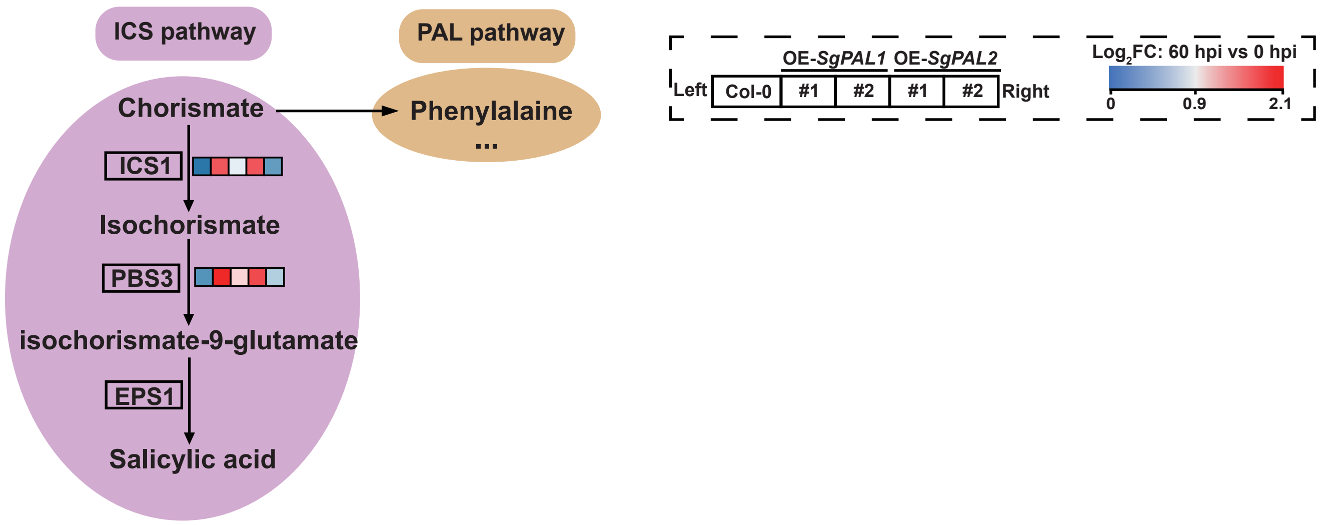

Fig S6. The 2 DEGs in the salicylic acid biosynthesis in Col-0, *SgPAL1* and *SgPAL2* overexpression plants after *C. gloeosporioides* infection. Data represent the mean of three biological replicates. ICS, isochorismate synthase; PBS3, avrPphB susceptible 3; EPS1, enhanced pseudomonas susceptibility 1.

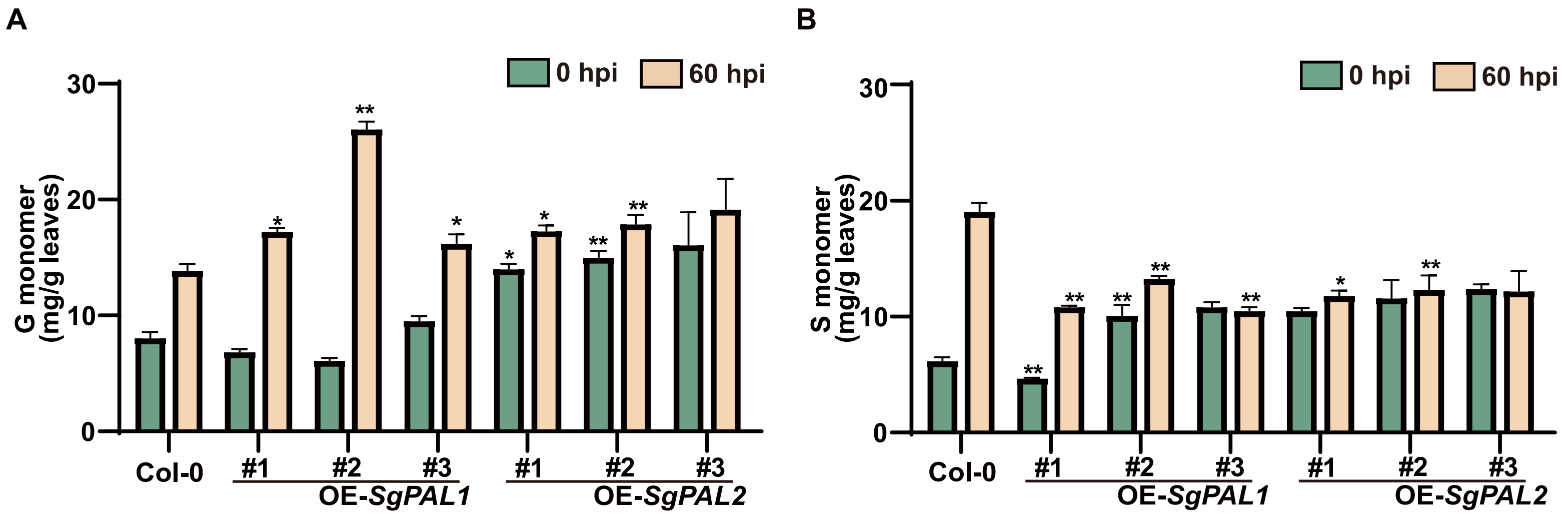

Fig. S7. Lignin monomer in Col-0, *SgPAL1* and *SgPAL2* overexpression plants before and after *C. gloeosporioides* infection. The content of G monomer (A) and S monomer (B). Data are the mean ± standard error of three independent biological replicates, and asterisks indicate significant differences (*$P < 0.05$, **$P < 0.01$) in the overexpression lines compared to Col-0 determined using the t-test.
