# Supplementary material for: SgPAL1/2 confers anthracnose resistance in Stylosanthes guianensis by modulating lignin content and monomer ratios
Source: BMC Plant Biol. 2025 Dec 24;25:1727. doi: 10.1186/s12870-025-07720-2 (PMC12729147; doi:10.1186/s12870-025-07720-2)
Supplement: Supplementary file 3 — Supplementary Material 3. [file 12870_2025_7720_MOESM3_ESM.docx]

**Supplementary Material 3**

**Genetic diversity analysis and core collection establishment in *S. guianensis* germplasm**

The characterization of genetic diversity in germplasm resources is fundamental for identifying key disease resistance genes. Therefore, we conducted comprehensive phenotypic and molecular analyses on 237 *S. guianensis* accessions. Phenotypic evaluation of 22 agronomic traits revealed a diversity index range of 0.049-1.171, with seed coat color showing the highest variation and leaf type exhibiting the lowest polymorphism (Table S6 and S7). Molecular characterization using 30 SSR markers detected 118 alleles, with mean values of 1.312 for effective allele number (Ne), 0.360 for Nei's genetic diversity index (h), and 0.700 for Shannon's diversity index (I) (Table S8). These diversity parameters significantly exceeded those previously reported for six *Stylosanthes* species [51], indicating broader genetic variation in our germplasm panel.

Population structure analysis identified five distinct subpopulations (I-V), with 83.1% of accessions showing strong population assignment (Q>0.8) and only 3.3% exhibiting admixture (Q<0.5) (Fig. S8A and B). UPGMA clustering revealed clear phylogenetic relationships among subpopulations, particularly demonstrating close genetic ties between branches B, C, and D, while branches A and E corresponded to subpopulations I and V, respectively (Fig. S8B).

To facilitate efficient utilization of these genetic resources, we established a core collection comprising 28 accessions (15% of total) through integrated phenotypic and SSR data analysis using Powercore and Core Hunter software (Fig. S8C and Table S1). Validation tests confirmed the representativeness of this core set, with homogeneity of variance maintained for 20 of 22 traits (excluding inflorescence type and seed coat color). The core collection retained over 78.57% of both phenotypic and molecular diversity (Tables S9and S10) and showed uniform distribution within the original germplasm in UPGMA and principal coordinate analyses (Fig. S8D). This well-characterized core collection provides an optimal resource for subsequent disease resistance gene mining in stylo.

**Material and methods**

**Plant material and growth conditions**

The 237 accessions of stylo (*S. guianensis*) for phenotypic observation and SSR detection were planted in the experimental station of the Tropical Crops Genetic Resources Institute & National Key Laboratory for Tropical Crop Breeding, Chinese Academy of Tropical Agricultural Sciences (CATAS). The experimental field soil is classified as granite red soil, belonging to the tropical monsoon climate type. The seeds were soaked in hot water (80°C) for 3 min, followed by a 3-day germination phase as previously described. Seedlings were then relocated to pots containing vermiculite and organic soil (2:1, v/v), watered by sufficient half-strength Hoagland's nutrient solution, and grown under greenhouse condition (28°C) for 30 days [22].

**Analysis of genetic diversity, population structure and phylogenetic tree in stylo populations**

One-month-old stylo seedlings were transplanted into plots, with 15 plants per plot, spaced 20 cm apart. The inter-row spacing between different materials was 2 meters, and the inter-column spacing was 1.5 meters, with routine field management practices followed. Based on the determination guidelines, 18 quality traits and 4 quantitative traits were investigated. For all traits, the genetic diversity index was calculated as H'= -ΣP_i_LnP_i_, where P_i_ denotes the probability of the occurrence of the i-th level of a given trait. In addition, frequency distribution was determined for 18 quality traits. According to Ding’s research [52], DNA was extracted from latest unfolded leaves from five plants of each accession during the vegetative growth phase, followed by SSR analysis using 30 pairs of SSR primers with high polymorphism were synthesized by Sangon Biotech (Shanghai, China). Genetic diversity parameters for each marker were calculated using GenAlex 6.5 and Popgene v.32 software, including number of alleles (Na), number of effective alleles (Ne), observed heterozygosity (Ho), expected heterozygosity (He), Nei genetic diversity index (h) and Shannon diversity index (I). The polymorphism information content (PIC) of simple sequence repeats (SSRs) was determined by Cervus 2.0 software.

The genetic composition of the populations was evaluated using STRUCTURE software, which calculated the optimal number of genetic clusters. The population structure was determined based on the Bayesian model, and the maximum likelihood value was generated for the simulation results of each K-value [53]. The burn-in period and MCMC iterations were set at 10,000 and 100, 000, respectively. The range of grouping values (K-values) for the subgroups was from 2 to 14, with simulations for each K-value repeated five times. Subsequently, data were uploaded to the Structure Selector (https://lmme.ac.cn/StructureSelector/) website to calculate the most suitable K-value based on the ΔK method. Population structure was determined based on Q-matrix data. Genetic similarity coefficients were calculated based on the molecular marker matrix, and a clustering tree was constructed by unweighted class average (UPGMA) clustering method in NTSYSPC2.10e software. The genetic structure map and evolutionary tree were visualized using the iTOL website (<https://itol.embl.de/>).

**Construction of core collection and evaluation of hereditary diversity**

The core collection with a sampling proportion of 15% was constructed by R package (Core hunnter) and Powercore V2 software. The common collection selected by two software was obtained based on TBtools Wayne diagram analysis. The means and homogeneity of variances for each trait in the original and core collections were compared using the Newman-Keuls procedure and Levene's test, respectively [54-56]. The homogeneity of distribution for all qualitative and quantitative traits was further examined using the chi-square *ꭕ2*-test. The genetic diversity parameters of the core collection were calculated by GenAlex 6.5 and Popgene v.32 software, and the retention ratio (core/original germplasms) was calculated sequentially. PCoA was performed using R package (stats 3.5.1.)

**References**

[22] Hoagland DR, Arnon DI. The water culture method for growing plants without soil. California Agric. Exp. Station Circular. 1950;36-39. <https://doi.org/10.1016/S0140-6736(00)73482-9>.

[51] Huang C, Liu G, Bai C. Polymorphism analysis in identification of genetic variation and relationships among *Stylosanthes* species. 3 Biotech. 2017;7:39. https://doi.org/10.1007/s13205-017-0705-x.

[52] Ding X, Jia Q, Luo X, Zhang L, Cong H, Liu G, et al. Development and characterization of expressed sequence tag-derived simple sequence repeat markers in tropical forage legume *Stylosanthes guianensis* (Aubl.) Sw. Mol. Breed. 2015;35:202. https://doi.org/10.1007/s11032-015-0370-x.

[53] Evanno G, Regnaut S, Goudet J. Detecting the number of clusters of individuals using the software STRUCTURE: a simulation study. Mol. Ecol. 2005;14:2611-2620. https://doi.org/10.1111/j.1365-294X.2005.02553.x.

[54] Newman D. The distribution of range in samples from a normal population expressed in terms an independent estimate of standard deviation. Biometrika. 1939;31:20-30. https://doi.org/10.2307/2334973.

[55] Keuls M. The use of the “studentized range” in connection with an analysis of variance. Euphytica. 1952;1:112-122. https://doi.org/10.1007/BF01908269.

[56] Levene H. Robust Tests for Equality of Variances. In: Olkin, I, editors. Contributions to probability and statistics: essays in honor of harold hotelling. Stanford University Press, Palo Alto; 1960. p. 278-292.


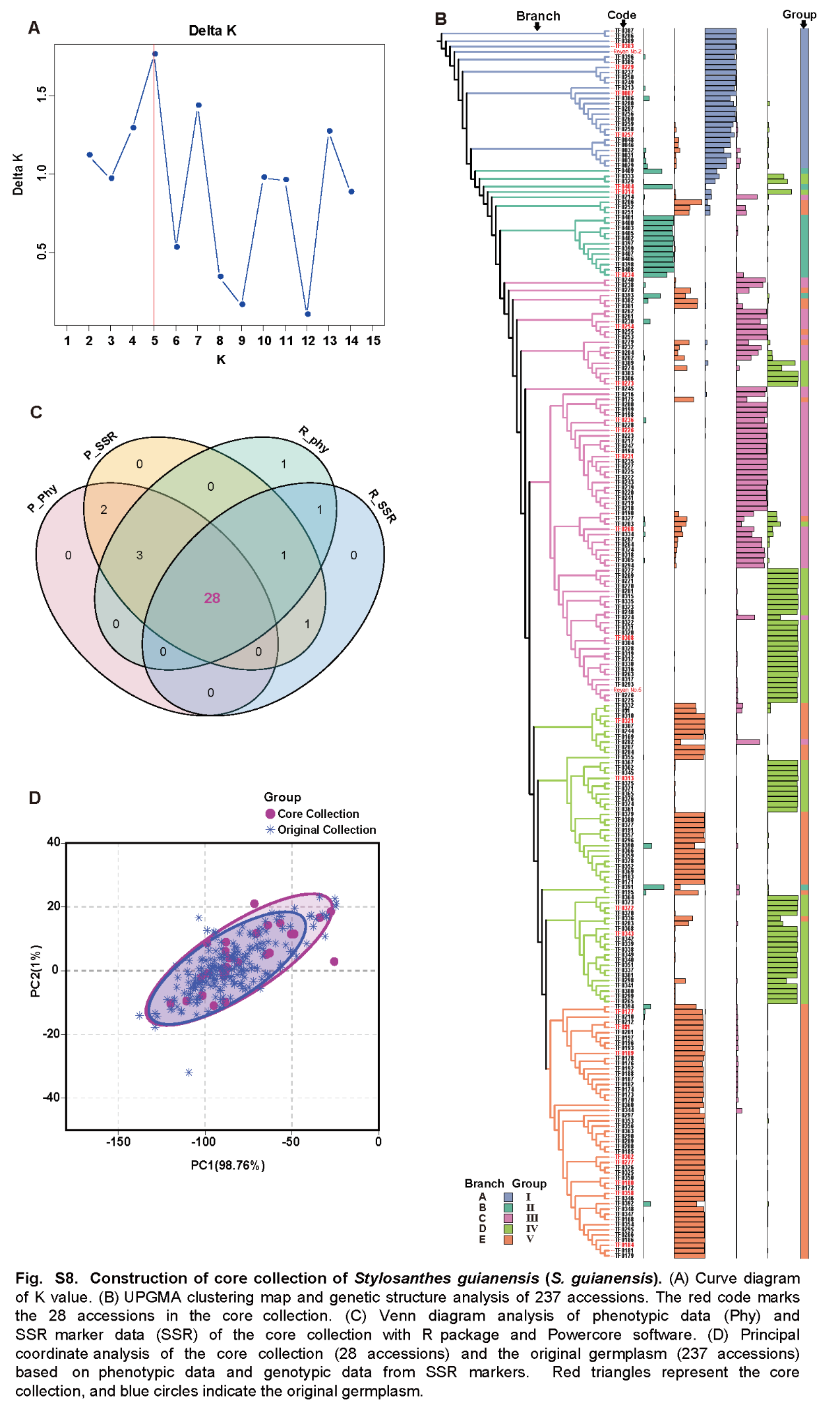


**Table S6 Diversity analysis for 18 quality traits of 237 *Stylosanthes guianensis* accessions**

| **Traits** | **Genetic diversity (H')** | **Frequency distribution**^1)^ | | | | |
| --- | --- | --- | --- | --- | --- | --- |
|  |  | **1** | **2** | **3** | **4** | **5** |
| Stem growth habits | 0.347 | 0.030 | 0.924 | 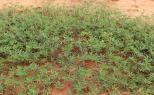0.034 | 0.013 | — |
|  |  | 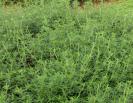 | 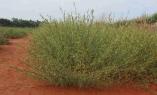 |  | 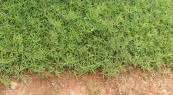 |  |
| Stem color | 0.133 | 0.970 | 0.030 | — | — | — |
|  |  | 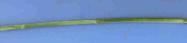 | 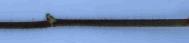 |  |  |  |
| Stem hair type | 0.342 | 0.835 | 0.013 | 0.013 | 0.118 | 0.021 |
|  |  | 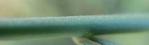 | 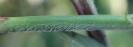 | 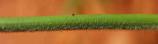 | 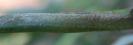 | 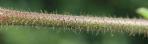 |
| Leaf type | 0.049 | 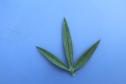0.008 | 0.992 | — | — | — |
|  |  |  | 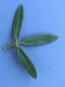 |  |  |  |
| Leaf color | 0.325 | 0.013 | 0.916 | 0.072 | — | — |
|  |  | 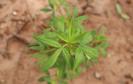 | 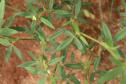 | 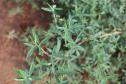 |  |  |
| Leaflet shape | 0.279 | 0.920 | 0.080 | — | — | — |
|  |  | 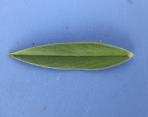 | 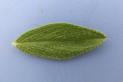 |  |  |  |
| Leaf surface hair | 0.896 | 0.414 | 0.072 | 0.515 | — | — |
|  |  | 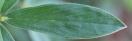 | 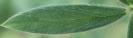 | 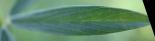 |  |  |
| Leaf dorsiventral hair | 0.990 | 0.540 | 0.165 | 0.295 | — | — |
|  |  | 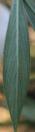 | 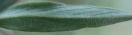 | 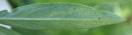 |  |  |
| Stipule hair | 1.018 | 0.422 | 0.422 | 0.156 | — | — |
|  |  | 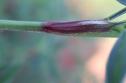 | 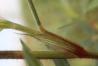 | 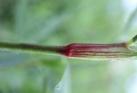 |  |  |
| Inflorescence type | 0.380 | 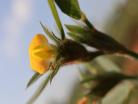0.127 | 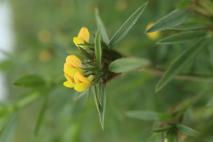0.873 | — | — | — |
|  |  |  |  |  |  |  |
| Foret insertion modality | 0.086 | 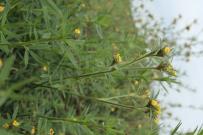0.983 | 0.017 | — | — | — |
|  |  |  | 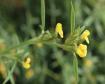 |  |  |  |
| Banner stripe | 0.939 | 0.008 | 0.228 | 0.629 | 0.135 | — |
|  |  | 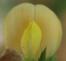. | 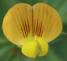 | 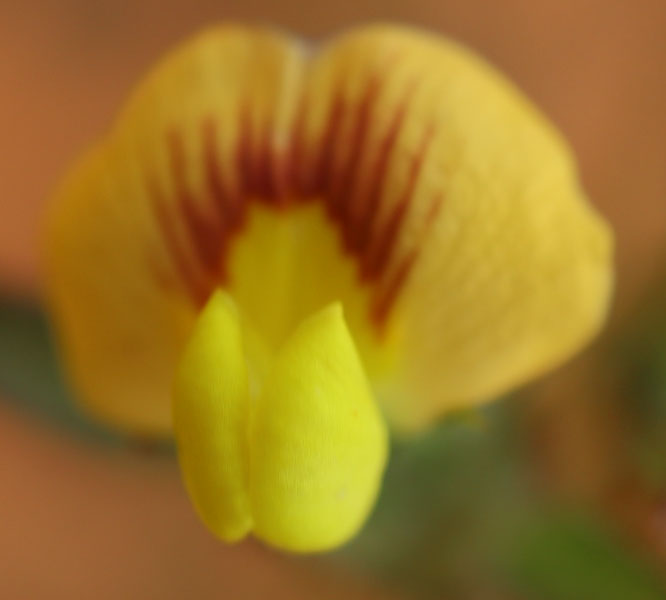 | 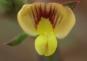 |  |
| Banner color | 0.882 | 0.072 | 0.034 | 0.203 | 0.692 | — |
|  |  | 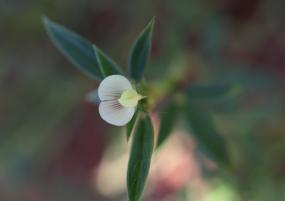 | 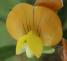 | 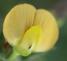 | 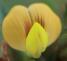 |  |
| Carene tip shape | 0.068 | 0.987 | 0.013 | — | — | — |
|  |  | 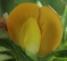 | 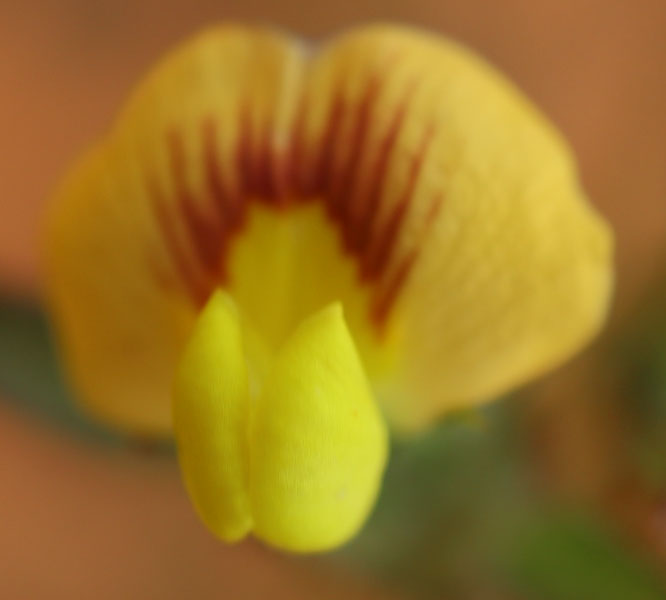 |  |  |  |
| Pod shape | 0.420 | 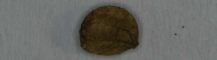0.110 | 0.004 | 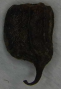0.008 | 0.878 | — |
|  |  |  | 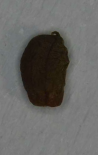 |  | 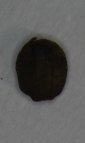 |  |
| Pod beak shape | 0.736 | 0.169 | 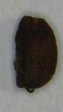0.743 | 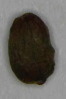0.089 | — | — |
|  |  | 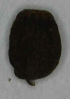 |  |  |  |  |
| Seed coat color | 1.171 | 0.135 | 0.608 | 0.148 | 0.042 | 0.068 |
|  |  | 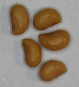 | 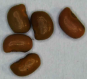 | 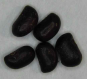 | 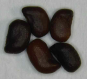 | 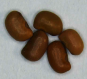 |
| Seed shape | 0.388 | 0.911 | 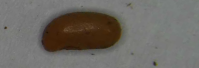0.042 | 0.034 | 0.013 | — |
|  |  | 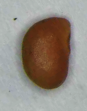 |  | 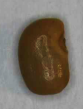 | 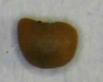 |  |

^1)^ Frequency distribution of 1, 2, 3, 4, 5 are same with classification of the traits in Table A.2.

**Table S7 Diversity analysis for 4 quantitative quality traits of 237 *S. guianensis* accessions**

| **Traits** |  | **Mean** | **SD**^1)^ | **Max**^2)^ | **Min**^3)^ | **VR**^4)^ | **CV/%**^5)^ | **H'**^6)^ |
| --- | --- | --- | --- | --- | --- | --- | --- | --- |
| Petiole length（mm） | 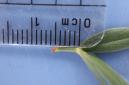 | 6.96 | 1.53 | 11.81 | 2.94 | 8.87 | 21.96 | 0.56 |
| Leaf length（mm） | 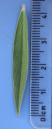 | 32.44 | 6.18 | 54.20 | 9.63 | 44.57 | 19.05 | 0.68 |
| Leaf width（mm） | 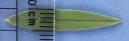 | 6.44 | 1.11 | 10.91 | 3.28 | 7.63 | 17.20 | 0.81 |
| Plant height（cm） | 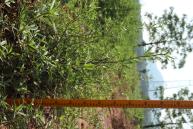 | 76.69 | 23.06 | 131.84 | 14.00 | 117.84 | 30.07 | 0.60 |

^1)^SD, standard deviation.

^2)^Max, maximum.

^3)^Mix, minimum.

^4)^VR, variance range.

^5)^CV, coefficient of variation.

^6)^H’, Genetic diversity.

**Table S8 SSR genetic diversity analysis of 237 *S. guianensis* accessions**

| **Primer** | **Na**^1)^ | **Ne**^2)^ | **Ho**^3)^ | **He**^4)^ | **h**^5)^ | **I**^6)^ | **PIC**^7)^ |
| --- | --- | --- | --- | --- | --- | --- | --- |
| RM3 | 4 | 1.168 | 0.017 | 0.144 | 0.498 | 0.860 | 0.140 |
| RM5 | 4 | 2.993 | 0.013 | 0.667 | 0.498 | 0.860 | 0.594 |
| RM14 | 4 | 1.180 | 0.059 | 0.153 | 0.208 | 1.208 | 0.147 |
| RM22 | 4 | 2.026 | 0.004 | 0.508 | 0.219 | 1.235 | 0.391 |
| RM23 | 2 | 1.825 | 0.148 | 0.453 | 0.153 | 0.633 | 0.350 |
| RM47 | 5 | 1.148 | 0.017 | 0.129 | 0.114 | 0.726 | 0.126 |
| RM48 | 3 | 1.021 | 0.004 | 0.021 | 0.445 | 0.777 | 0.021 |
| RM52 | 4 | 1.066 | 0.013 | 0.062 | 0.445 | 0.777 | 0.061 |
| RM63 | 3 | 1.127 | 0.002 | 0.113 | 0.246 | 0.505 | 0.108 |
| RM65 | 4 | 1.303 | 0.215 | 0.233 | 0.257 | 0.518 | 0.215 |
| RM77 | 4 | 1.066 | 0.021 | 0.062 | 0.241 | 0.497 | 0.061 |
| RM92 | 5 | 1.613 | 0.110 | 0.381 | 0.130 | 0.729 | 0.363 |
| RM113 | 4 | 1.173 | 0.013 | 0.148 | 0.967 | 0.057 | 0.143 |
| RM116 | 3 | 1.600 | 0.021 | 0.376 | 0.967 | 0.057 | 0.309 |
| RM138 | 4 | 1.168 | 0.008 | 0.144 | 0.495 | 0.840 | 0.141 |
| RM145 | 4 | 1.113 | 0.013 | 0.102 | 0.496 | 0.800 | 0.100 |
| RM207 | 4 | 1.177 | 0.009 | 0.151 | 0.253 | 1.038 | 0.142 |
| RM243 | 4 | 1.233 | 0.025 | 0.190 | 0.253 | 1.038 | 0.184 |
| RM301 | 3 | 1.232 | 0.008 | 0.189 | 0.017 | 0.089 | 0.182 |
| RM302 | 4 | 1.157 | 0.018 | 0.136 | 0.321 | 0.928 | 0.132 |
| RM366 | 4 | 1.114 | 0.013 | 0.102 | 0.478 | 0.805 | 0.100 |
| RM369 | 4 | 1.323 | 0.148 | 0.245 | 0.499 | 0.815 | 0.233 |
| RM392 | 5 | 1.349 | 0.043 | 0.259 | 0.039 | 0.701 | 0.247 |
| RM417 | 5 | 1.042 | 0.018 | 0.041 | 0.265 | 1.233 | 0.040 |
| RM424 | 4 | 1.449 | 0.283 | 0.311 | 0.792 | 0.150 | 0.279 |
| RM444 | 5 | 1.090 | 0.017 | 0.083 | 0.792 | 0.127 | 0.082 |
| RM448 | 3 | 1.143 | 0.030 | 0.126 | 0.284 | 1.142 | 0.123 |
| RM469 | 4 | 1.052 | 0.000 | 0.050 | 0.288 | 1.143 | 0.049 |
| RM474 | 4 | 1.171 | 0.030 | 0.147 | 0.071 | 0.350 | 0.140 |
| RM500 | 4 | 1.226 | 0.021 | 0.189 | 0.071 | 0.350 | 0.182 |
|  |  |  |  |  |  |  |  |
| Mean | 3.933 | 1.312 | 0.045 | 0.197 | 0.360 | 0.700 | 0.180 |

^1)^Na, number of alleles.

^2)^Ne, number of effective alleles.

^3)^Ho, observed heterozygosity.

^4)^He, expected heterozygosity.

^5)^h, Nei's genetic diversity index.

^6)^I, Shannon's diversity index.

^7)^PIC, polymorphism information content.

| **Table S9 Comparison of mean (± SE), variance, frequency of distribution and Shannon Weaver diversity index in the original and core collection for 22 traits** | | | | | | | | | | | | | | | | | | | | | |
| --- | --- | --- | --- | --- | --- | --- | --- | --- | --- | --- | --- | --- | --- | --- | --- | --- | --- | --- | --- | --- | --- |
| **Traits** | **Means**^1)^ | | | | |  | **Variance**^1)^ | | | |  | | **Frequency of distribution** | | | |  | **Shannon-Weaver diversity index** | | |  |
|  | **Original collection** | | | **Core collection** | **Differences**^2)^ |  | **Original collection** | **Core collection** | **F value** | ***P*** ^3)^ | |  | | **df** | ***ꭕ^2^*** | ***P*** ^3)^ |  | **Original collection** | **Core collection** | **Retention ratio (%)** ^4)^ |  |
| **Qualitative traits** | |  | |  |  |  |  |  |  |  | |  | |  |  |  |  |  |  |  |  |
| Stem growth habits | 2.03±0.22 | | | 2.07±0.71 | NS^2)^ |  | 0.11 | 0.14 | 1.74 | 0.190 | |  | | 3 | 3.757 | 0.289 |  | 0.347 | 0.582 | 167.88 |  |
| Stem color | 1.03±0.01 | | | 1.04±0.04 | NS |  | 0.03 | 0.04 | 0.13 | 0.720 | |  | | 1 | 0.033 | 0.857 |  | 0.133 | 0.328 | 246.02 |  |
| Stem hair type | 1.48±0.07 | | | 1.61±0.24 | NS |  | 1.24 | 1.58 | 1.02 | 0.313 | |  | | 4 | 1.674 | 0.795 |  | 0.342 | 0.745 | 217.63 |  |
| Leaf type | 1.99±0.01 | | | 1.96±0.04 | NS |  | 0.01 | 0.04 | 6.52 | 0.011 | |  | | 1 | 1.664 | 0.197 |  | 0.049 | 0.328 | 672.55 |  |
| Leaf color | 2.06±0.02 | | | 2.00±0.05 | NS |  | 0.08 | 0.07 | 1.59 | 0.208 | |  | | 2 | 1.365 | 0.505 |  | 0.325 | 0.443 | 136.33 |  |
| Leaflet shape | 1.08±0.02 | | | 1.07±0.05 | NS |  | 0.07 | 0.07 | 0.11 | 0.745 | |  | | 1 | 0.026 | 0.871 |  | 0.279 | 0.406 | 145.3 |  |
| Leaf surface hair | 2.10±0.06 | | | 2.14±0.18 | NS |  | 0.92 | 0.87 | 1.75 | 0.188 | |  | | 2 | 1.804 | 0.406 |  | 0.896 | 0.962 | 107.35 |  |
| Leaf dorsiventral hair | 1.76±0.06 | | | 1.96±0.17 | NS |  | 0.78 | 0.78 | 0.72 | 0.397 | |  | | 2 | 2.405 | 0.300 |  | 0.99 | 1.029 | 103.98 |  |
| Stipule hair | 1.73±0.05 | | | 1.89±0.14 | NS |  | 0.51 | 0.54 | 0.39 | 0.531 | |  | | 2 | 1.243 | 0.537 |  | 1.018 | 1.006 | 98.85 |  |
| Inflorescence type | 1.87±0.02 | | | 1.64±0.09 | * |  | 0.11 | 0.24 | 24.65 | 0.000 | |  | | 1 | 0.238 | 0.626 |  | 0.38 | 0.704 | 185.38 |  |
| Foret insertion modality | | 1.02±0.01 | | 1.04±0.04 | NS |  | 0.02 | 0.04 | 1.86 | 0.173 | |  | | 1 | 0.48 | 0.488 |  | 0.086 | 0.328 | 382.5 |  |
| Banner stripe | 2.89±0.04 | | | 3.04±0.11 | NS |  | 0.39 | 0.33 | 1.11 | 0.292 | |  | | 3 | 1.501 | 0.682 |  | 0.939 | 0.853 | 90.82 |  |
| Banner color | 3.51±0.06 | | | 3.64±0.13 | NS |  | 0.75 | 0.46 | 2.27 | 0.133 | |  | | 3 | 1.713 | 0.634 |  | 0.882 | 0.745 | 84.52 |  |
| Carene tip shape | 1.01±0.01 | | | 1.04±0.04 | NS |  | 0.01 | 0.04 | 3.48 | 0.063 | |  | | 1 | 0.895 | 0.344 |  | 0.068 | 0.328 | 482.44 |  |
| Pod shape | 3.65±0.06 | | | 3.68±0.18 | NS |  | 0.90 | 0.89 | 0.05 | 0.819 | |  | | 3 | 0.363 | 0.948 |  | 0.42 | 0.469 | 111.47 |  |
| Pod beak shape | 1.92±0.03 | | | 2.00±0.10 | NS |  | 0.25 | 0.30 | 0.10 | 0.758 | |  | | 2 | 0.91 | 0.635 |  | 0.736 | 0.814 | 110.53 |  |
| Seed coat color | 2.30±0.06 | | | 2.61±0.27 | NS |  | 0.97 | 2.03 | 11.52 | 0.001 | |  | | 4 | 8.393 | 0.078 |  | 1.171 | 1.189 | 101.49 |  |
| Seed shape | 1.15±0.03 | | | 1.29±0.14 | NS |  | 0.27 | 0.58 | 5.76 | 0.017 | |  | | 3 | 1.93 | 0.587 |  | 0.388 | 0.634 | 163.4 |  |
| **Mean ± SE** |  | | |  |  |  |  |  |  |  | |  | |  |  |  |  | 0.525±0.09 | 0.661±0.07 |  |  |
| **Quantitative traits** | | |  |  |  |  |  |  |  |  | |  | |  |  |  |  |  |  |  |  |
| Petiole length（mm） | | 6.69±0.10 | | 6.77±0.32 | NS |  | 2.33 | 2.91 | 0.34 | 0.561 | |  | | 2 | 0.443 | 0.801 |  | 0.561 | 0.668 | 119.06 |  |
| Leaf length（mm） | | 32.44±0.40 | | 31.84±1.32 | NS |  | 38.19 | 48.98 | 0.10 | 0.749 | |  | | 2 | 0.574 | 0.751 |  | 0.675 | 0.706 | 104.51 |  |
| Leaf width（mm） | | 6.44±0.07 | | 6.38±0.24 | NS |  | 1.23 | 1.68 | 0.74 | 0.392 | |  | | 2 | 2.224 | 0.329 |  | 0.808 | 0.956 | 118.23 |  |
| Plant height（cm） | | 76.69±1.50 | | 69.73±4.74 | NS |  | 531.70 | 629.64 | 1.11 | 0.294 | |  | | 2 | 3.388 | 0.184 |  | 0.597 | 0.469 | 78.57 |  |
| **Mean ± SE** | |  | |  |  |  |  |  |  |  | |  | |  |  |  |  | 0.660±0.33 | 0.700±0.35 |  |  |
| **Mean over-all ± SE** | |  | |  |  |  |  |  |  |  | |  | |  |  |  |  | 0.550±0.07 | 0.668±0.06 |  |  |
| ^1)^Differences between means of original and core collection tested by Newman-Keuls test and variance homogeneity by Levene's test.  ^2)^NS and * indicate non-significant or significant differences, respectively, at *P*=0.05.  ^3)^*P* is the probability level of significance.  ^4)^Retention ratio (%) = Core collection/ Original collection * 100%. | | | | | | | | | | | | | | | | | | | | |  |

**Table S10 The comparison of genetic diversity based on molecular data between core collection and original collection in *S. guianensis***

| **Population** | **total** | **Na**^1)^ | **Ne**^2)^ | **h**^3)^ | **I**^4)^ | **Ho**^5)^ | **He**^6)^ | **Ht**^7)^ | **Hs**^8)^ | **Gst**^9)^ | **PIC**^10)^ |
| --- | --- | --- | --- | --- | --- | --- | --- | --- | --- | --- | --- |
| Core collection | 112 | 3.733 | 1.434 | 0.366 | 0.715 | 0.053 | 0.266 | 0.592 | 0.328 | 1.997 | 0.238 |
|  |  |  |  |  |  |  |  |  |  |  |  |
| Original collection | 118 | 3.933 | 1.312 | 0.360 | 0.700 | 0.045 | 0.197 | 0.609 | 0.311 | 1.955 | 0.180 |
|  |  |  |  |  |  |  |  |  |  |  |  |
| Retention ratio (%)^11)^ | 94.92 | 94.92 | 109.32 | 101.69 | 102.19 | 117.70 | 135.05 | 97.29 | 105.40 | 102.14 | 132.61 |

^1)^Na, number of alleles.

^2)^Ne, number of effective alleles.

^3)^h, Nei's genetic diversity index.

^4)^I, Shannon's diversity index.

^5)^Ho, observed heterozygosity.

^6)^He, expected heterozygosity.

^7)^Ht, total population genetic diversity.

^8)^Hs, intra-population genetic diversity.

^9)^Gst, genetic differentiation index between populations.

^10)^PIC, polymorphism information content.

^11)^Retention ratio (%) = Core collection/ Original collection * 100%.
